# Toward the design of persuasive systems for a healthy workplace: a real-time posture detection

**DOI:** 10.3389/fdata.2024.1359906

**Published:** 2024-06-17

**Authors:** Grace Ataguba, Rita Orji

**Affiliations:** Department of Computer Science, Dalhousie University, Halifax, NS, Canada

**Keywords:** persuasive technology, healthy workplace, posture, machine learning, YOLO-V3, convolutional neural networks

## Abstract

Persuasive technologies, in connection with human factor engineering requirements for healthy workplaces, have played a significant role in ensuring a change in human behavior. Healthy workplaces suggest different best practices applicable to body posture, proximity to the computer system, movement, lighting conditions, computer system layout, and other significant psychological and cognitive aspects. Most importantly, body posture suggests how users should sit or stand in workplaces in line with best and healthy practices. In this study, we developed two study phases (pilot and main) using two deep learning models: convolutional neural networks (CNN) and Yolo-V3. To train the two models, we collected posture datasets from creative common license YouTube videos and Kaggle. We classified the dataset into comfortable and uncomfortable postures. Results show that our YOLO-V3 model outperformed CNN model with a mean average precision of 92%. Based on this finding, we recommend that YOLO-V3 model be integrated in the design of persuasive technologies for a healthy workplace. Additionally, we provide future implications for integrating proximity detection taking into consideration the ideal number of centimeters users should maintain in a healthy workplace.

## 1 Introduction

The importance of persuasive technologies in influencing changes in human behavior is significant and cannot be overemphasized. Persuasive technologies have an impact on users' behavior and the choices they make (Rapoport, 2017; Orji et al., 2018; Darioshi and Lahav, 2021; Wang et al., 2023). As a result, persuasive technologies prioritize user-centered design, and they can assist users in leading a healthy lifestyle. Considering this, research has demonstrated the valuable roles these technologies play in preventing and aiding the management of illnesses (Schnall et al., 2015; Karppinen et al., 2016; Sonntag, 2016; Bartlett et al., 2017; Faddoul and Chatterjee, 2019; Fukuoka et al., 2019; Kim M. T. et al., 2019; Oyibo and Morita, 2021), promoting fitness and exercise (Bartlett et al., 2017; Schooley et al., 2021), and other significant ones (Jafarinaimi et al., 2005; Anagnostopoulou et al., 2019; Beheshtian et al., 2020).

The workplace, a location, setting, or environment where people engage in work, have recorded significant unhealthy practices, including bad posture, over the years (Nanthavanij et al., 2008; Ko Ko et al., 2020; Roy, 2020; van de Wijdeven et al., 2023). In the context of this study, we consider work-from-home (WFH) contexts, offices, and other spaces where computers are employed to be workplaces. Best workplace practices are significant for a healthy working style. These practices cover the need to ensure that computer users maintain the right posture, follow the right movement practices, take regular breaks from computer systems, ensure they have proper lighting conditions, adhere to computer system layout, and other significant psychological and cognitive aspects. Poor workplace practices can lead to various health issues, such as repetitive strain injuries, eyestrain, and postural problems (Ofori-Manteaw et al., 2015; Workineh and Yamaura, 2016; Alaydrus and Nusraningrum, 2019). Research has shown that over 70% of stress, neck injuries, other types of sprains and pains (for example, arm sprains and back pain), and stress are work-related (Tang, 2022). This study presents the design of a persuasive system based on the best posture practices. In addition, this study presents implications for designing persuasive systems based on their proximity to computer system requirements.

Machine learning, a subfield of artificial intelligence (AI), deals with developing models. These models assist computers in learning and detecting patterns of objects in the real world (Mahesh, 2020; Sarker, 2021). Hence, machine learning has contributed to several studies that have significantly detected patterns in human behaviors (Cheng et al., 2017; Krishna et al., 2018; Xu et al., 2019; Chandra et al., 2021; Jupalle et al., 2022; Cob-Parro et al., 2023), human emotions (Jaiswal and Nandi, 2020; Gill and Singh, 2021), and health-related behaviors (Reddy et al., 2018; Mujumdar and Vaidehi, 2019; Ahmad et al., 2021). In this study, we leverage the opportunity of machine learning algorithms to design a persuasive system for detecting patterns of unhealthy postures and proximity to computers in workplaces.

**Figure 1:**
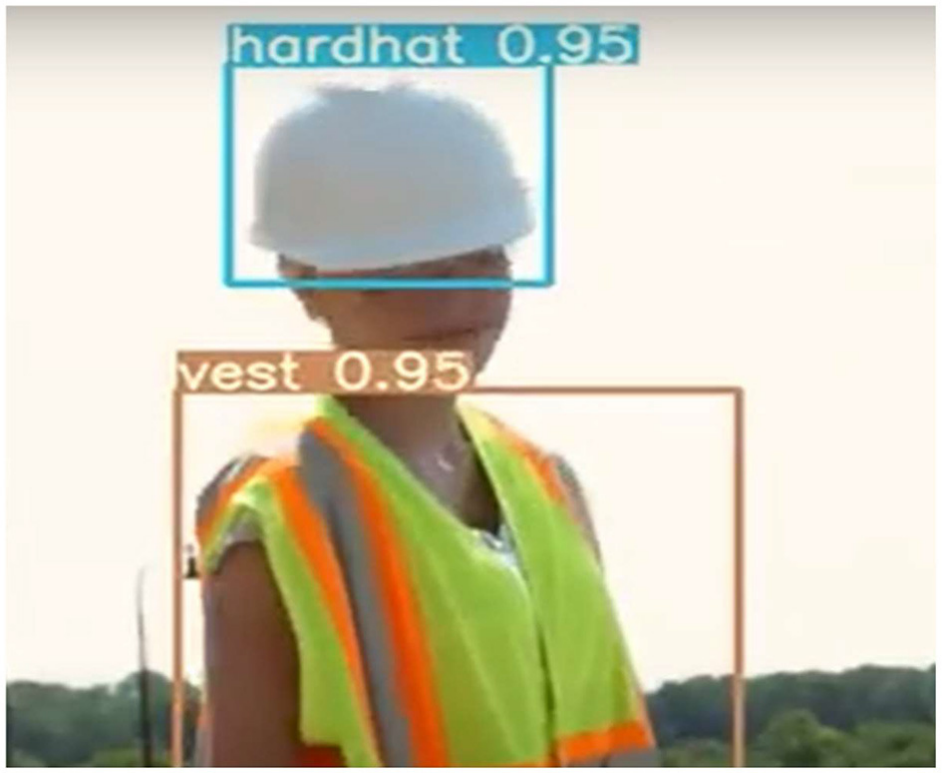
A YOLO-V3 detection on a sample image. Reproduced from “YOLOv3 on custom dataset,” YouTube, uploaded by “Aman Jain,” 22 July 2021, https://www.youtube.com/watch?v=D4RQ7Rkrass, Permissions: YouTube Terms of Service.

As part of persuasive technology's goal to provide users with real-time feedback on their actions (which, in turn, influences their behavior), we report on our experiment comparing the convolutional neural networks (CNN) and Yolo-V3 models. Research has shown the success of these models in real-time object detection (Tan et al., 2021; Alsanad et al., 2022). One of the significant drawbacks of CNN compared with Yolo-V3 from research is its requirement for a large number of training sets (Han et al., 2018). On the other hand, the Yolo-V3 model generates regions or boxes around objects and returns its accuracy values within these boxes. This implies that several boxes are marked within an object, and its performance can be implied from the confidence of predictions (Figure 1). For example, in Figure 1, the YOLO-V3 model predicted the hardhat with 95% confidence. Yolo-V3 and CNN work in real time by analyzing images extracted from frames per second and providing a consistent update as these images change.

Though we found significant studies in the application of persuasive systems to encourage computer users to take regular breaks from workplaces (Jafarinaimi et al., 2005; Reeder et al., 2010; Ludden and Meekhof, 2016; Ren et al., 2019), little is yet known about how they maintain the right posture before these regular breaks. Based on this limitation, the overarching goal of our study is to explore how people can be conscious of their unhealthy posture practices in workplaces (while sitting or standing). This connects with the main research question we seek to answer (RQ): RQ: Can we design persuasive computers to detect unhealthy posture practices (such as sitting and standing) in workplaces?

**Figure 2:**
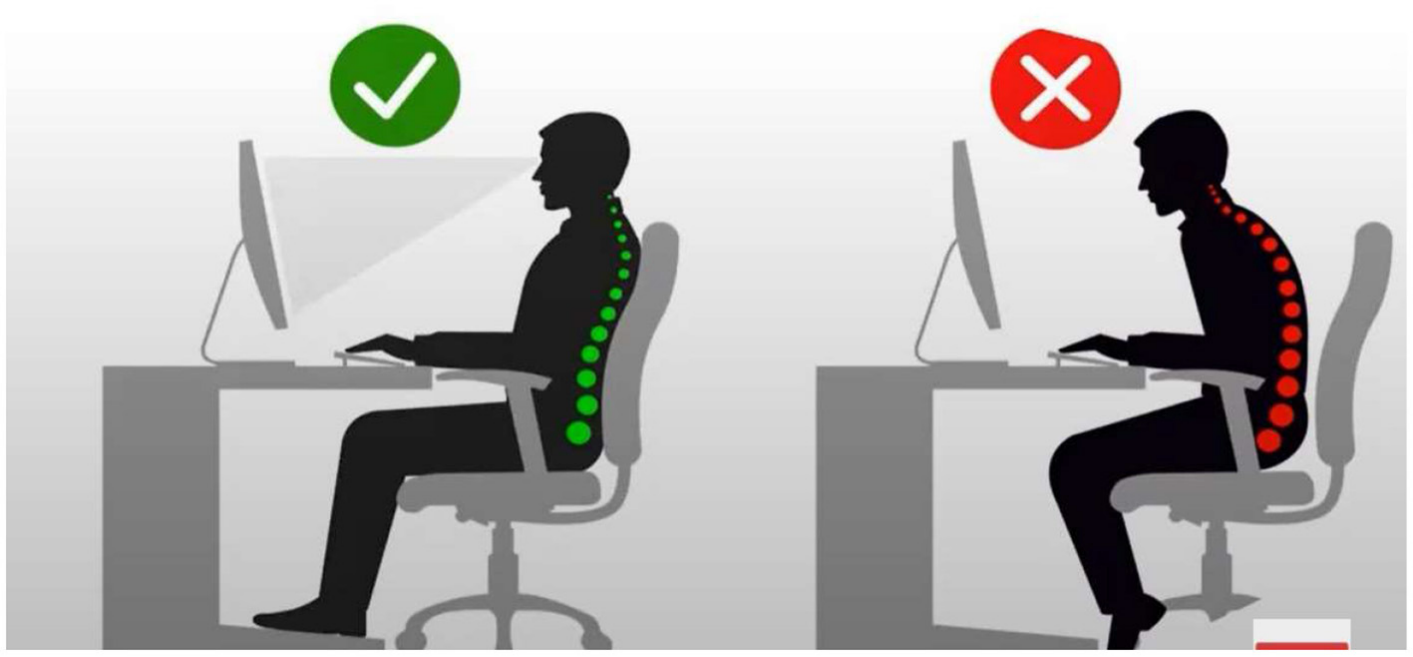
Correct ergonomics (L) and incorrect ergonomics (R) in a sitting workstation. Reproduced from “Computer Ergonomics,” YouTube, uploaded by “Pearls Classroom,” 5 October 2021, https://www.youtube.com/watch?v=XQTQ578wLzo, Permissions: YouTube Terms of Service.

**Figure 3:**
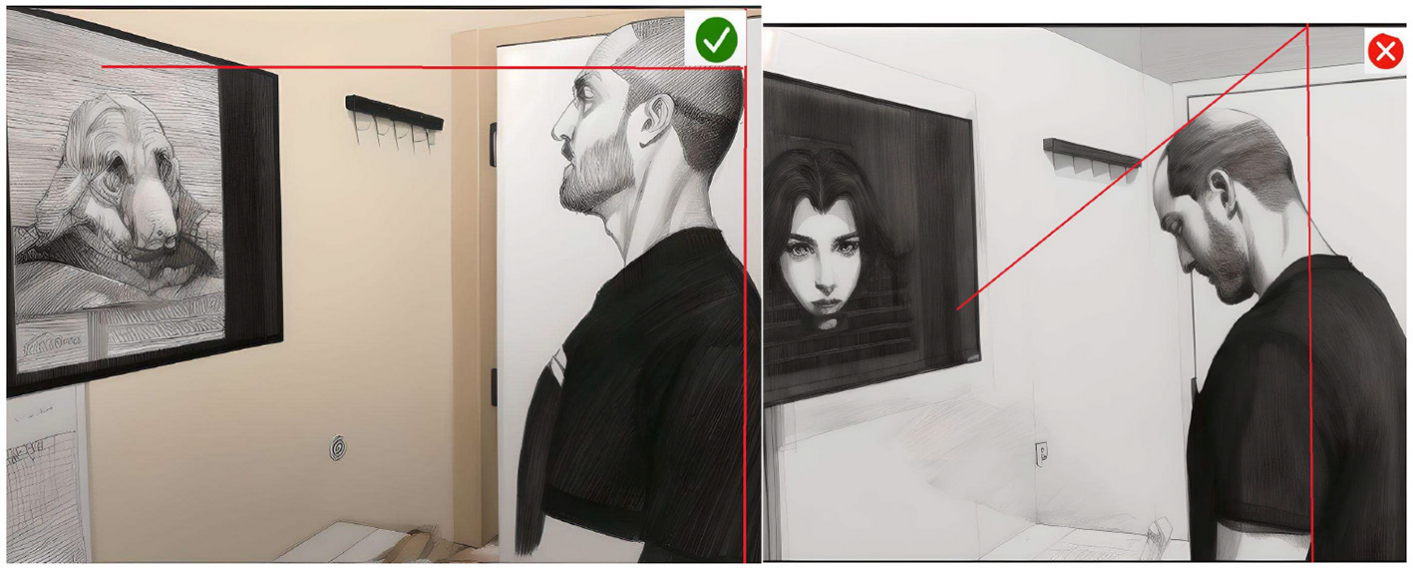
Edited scenes. Reproduced from “Libertyville IL neck pain—prevent bad posture with the right workstation,” YouTube, uploaded by “Functional Pain Relief,” 22 August 2018, https://www.youtube.com/watch?v=0M5C1BJdVsA, Permissions: YouTube Terms of Service.

People in workplaces have two types of posture positions: sitting and standing (Botter et al., 2016). The sitting position affords the computer user space to relax the back correctly on a chair (Figure 2, L). This, compared with the standing position, allows computer users to stand while using the computer system (Figure 3). It is significant to recall that before COVID-19, these workplaces were office spaces. However, most recently, after COVID-19, workplaces have extended to home spaces (Abdullah et al., 2020; Javad Koohsari et al., 2021). People now work from home, and the posture practices in these spaces have not been evaluated.

The scientific contributions of this research are in 4-folds:

1. Provision of ground truth posture datasets:

We are contributing ground-truth posture datasets for the research community to explore related concepts in the future. These datasets can be increased in future work to enhance the accuracy and effectiveness of future technological interventions. Hence, this contribution will support researchers and designers in developing more robust and context-aware persuasive technologies.

2. Implementation of deep learning models for posture detection:

We present the development and implementation of deep learning models for detecting the posture practices of computer users. These models leverage advanced techniques to interpret and classify diverse body positions, contributing to the evolving landscape of human–computer interaction. The models offer a technological solution to the challenge of real-time posture detection in the workplace. This contribution aligns with the forefront of research in machine learning and computer vision.

3. Real-time persuasive design for healthy workplace behavior:

We present a real-time persuasive design based on posture practices, thereby introducing a novel approach to promoting healthy workplace behavior. This contribution has practical implications for addressing issues related to sedentary work habits, discomfort, and potential health impacts associated with poor posture.

4. Integrating real-time feedback and persuasive elements:

Our design presents the potential and feasibility of persuasive technology to positively influence user behavior, fostering increased awareness and conscious efforts toward maintaining proper posture. This interdisciplinary contribution merges insights from computer science, psychology, and workplace health.

Collectively, these scientific contributions play a significant role in the advancement of knowledge in the fields of human–computer interaction, machine learning, and persuasive technology, with direct applications for improving workplace wellbeing and behavior. The rest of the study is structured as follows: First, we reviewed significant scholarly works on workplace practices, user health, and productivity; persuasive technologies and the workplace; machine learning and workplace practices; and accessibility technologies and healthy practices. Second, we present the methodology based on data collection and deep learning model deployment for the pilot study and the main study. Third, we report on the results of the pilot and main studies. In addition, we compare outcomes for deploying CNN and Yolo-V3 models toward persuasive, healthy workplace designs. Fourth, we present a discussion on the results from the pilot and main studies. Fifth, we report on the limitations of the study and present design recommendations to guide future research. Sixth, we conclude by summarizing the study and drawing an inference based on the results, limitations, and recommendations for future studies.

## 2 Related work

This section provides an in-depth exploration of related work comparing the relationship between workplace practices, user health and productivity, and other significant ones such as persuasive technologies and workplace practices, machine learning and workplace practices, and accessibility technologies and healthy practices.

### 2.1 Workplace practices, user health, and productivity

Workplace practices cover significant areas such as the proper chair and desk height, appropriate monitor placement, ergonomic keyboard and mouse usage, reduction of glare and reflection, importance of regular breaks, and promoting movement through sit-stand workstations (Dainoff et al., 2012; , 2023). Research has established a relationship between failing to adhere to good workplace practices and the consequences for computer users' health. These include the potential for musculoskeletal disorders, eye strain, and other common health issues related to prolonged computer use (Dainoff et al., 2012; Woo et al., 2016; Boadi-Kusi et al., 2022). According to Nimbarte et al. (2013), Shahidi et al. (2015), and Barrett et al. (2020), the force on the neck increases proportionately as the head angle tilts at a higher degree. The long-term impact of this, as shown in Table 1, is a spine damage risk.

**Table 1:** Relationship between the human head anatomy and exerted force leading to spine damage.^a^

**S/N**	**Degrees**	**Force (lb)**	**Spine damage risk level**
1.	0	10–12	Low or no risk
2.	15	27	Medium
3.	30	40	High
4.	60	50	Very high

^a^https://www.youtube.com/watch?v=0M5C1BJdVsA.

In addition, computer users' health is typically at risk due to repetitive stress injuries (Borhany et al., 2018; Mowatt et al., 2018; Iyengar et al., 2020; Roy, 2020; Steiger et al., 2021). Repetitive strain injury (RSI) is defined as “a chronic condition that develops because of repetitive, forceful, or awkward hand movements for prolonged periods leading to damage to muscles, tendons, and nerves of the neck, shoulder, forearm, and hand, which can cause pain, weakness, numbness, or impairment of motor control” (Sarla, 2019). This implies that computer use involving extended periods of typing and mouse use without proper ergonomics can increase the risk of RSIs. In addition, maintaining poor posture and not adhering to ergonomic requirements when setting up workstations can contribute to this risk. For example, Borhany et al. (2018) carried out a study to examine common musculoskeletal problems arising from the repetitive use of computers. They conducted a survey with 150 office workers and found that 67 of these workers suffer from repetitive stress injuries on the low back, neck, shoulder, and wrist/hand. In addition, they found that these injuries were caused by continuous use of computers without breaks, bad lighting, bad posture, and poorly designed ergonomics in offices. While it is typical that workplace tasks are characterized by repetitive tasks and actions, it has become imperative to design workplace technologies to support users in carrying out repetitive tasks without straining any part of the body (Moore, 2019; Johnson et al., 2020).

It is important to state that research has found the impact of computer users' health due to repetitive stress injuries and other related health issues on the productivity of users in workplaces. In other words, a well-designed workplace not only improves the user's comfort but also enhances work efficiency and overall job satisfaction (Pereira et al., 2019; Baba et al., 2021; Franke and Nadler, 2021). Pereira et al. (2019) examined 763 office workers in a 12-week study. They interpreted office productivity to be relative to absenteeism from work due to neck pain. The results from this study show that those exposed to healthy workplace practices and neck-specific exercise training had limited records of absenteeism. Pereira at al. reported that individuals with unhealthy workplace practices and limited access to health promotion information were more likely to be less productive, i.e., absent from work. Baba et al. (2021) conducted a study involving 50 newly employed staff in an organization. The staff was divided into experimental groups (with healthy workplace practices, e.g., comfortable computer desks) and control groups (with unhealthy workplace practices, such as less comfortable furniture). The study revealed a significant impact on the work productivity of the experimental group compared with the control groups (based on a *t*-test showing that t.cal = 0.08; t.tab = 1.71, where t.cal is the calculated *t*-test value and t.tab is the value of t in the distribution table).

While many organizations focus on employee training and sensitization programs for healthy workplace practices, limited research has been reported on workplace culture, employee training, computer workstation assessment, and the benefits of posture assessment tools. This study explores the potential of persuasive technologies for enhancing effective workplace posture practices. These technologies can serve as posture assessment tools, providing valuable feedback to organizations on the best ways to support their employees.

### 2.2 Persuasive technologies and the workplace

Persuasive technologies and workplace practices are two distinct areas of study and practice, but they intersect in designing user interfaces and technology systems that promote healthy workplace practices for technology users. Overall, this will enhance technology users' wellbeing and productivity. Research has explored persuasive technologies in relation to best workplace practices. This includes taking regular breaks (Jafarinaimi et al., 2005; Ludden and Meekhof, 2016; Ren et al., 2019), fitness apps (Mohadis et al., 2016; Ahtinen et al., 2017; Paay et al., 2022), feedback systems and wearable devices (Bootsman et al., 2019; Jiang et al., 2021), workstation movement (Min et al., 2015; Damen et al., 2020a,b), chair, desk, and monitor height adjustments (Kronenberg and Kuflik, 2019; Kronenberg et al., 2022), posture correction (Min et al., 2015; Bootsman et al., 2019; Kim M. T. et al., 2019), mouse/keyboard use and reduction of glare and reflection (Bailly et al., 2016), and other healthy work behaviors (Berque et al., 2011; Mateevitsi et al., 2014; Gomez-Carmona and Casado-Mansilla, 2017; Jiang et al., 2021; Brombacher et al., 2023; Haliburton et al., 2023; Robledo Yamamoto et al., 2023).

Table 2 summarizes closely related work on persuasive technologies with respect to workplace practices. We present discussions based on instances of workplace practices we listed previously. This includes taking regular breaks, fitness apps, feedback systems, workstation movement, chair, desk, monitor height adjustments, posture correction, mouse/keyboard use, reduction of glare and reflection, and other healthy practices. Jafarinaimi et al. (2005) developed sensor-based office chairs that encourage users to break away from their computers. Every 2 min, the chair slouches its position from upright to backward bend, signifying the need for computer users to take a break. In view of this, they experimented with a single user (55-year-old university staff). The results from the study showed how the sensor-based office chair greatly influenced the user's attitude to break away from their computer.

**Table 2:** Summary of research on persuasive technologies and workplace practices.

**S/N**	**References**	**Technology**	**Workplace practices covered**							
			**Chair and desk height**	**Monitor placement**	**Keyboard and mouse use**	**Reduction of glare and reflection**	**Regular breaks**	**Workstation movement**	**Posture correction**	**Other healthy practices**
1.	Haque et al. (2020)	Mobile App						□		
2.	Damen et al. (2020a)	Tangible						□		
3.	Damen et al. (2020b)	Phones, Tablets and Notebooks						□		
4.	Min et al. (2015)	Sensors						□	□	
5.	Ludden and Meekhof (2016)	Tangible					□			
6.	Jafarinaimi et al. (2005)	Tangible					□			
7	Kronenberg and Kuflik (2019)	Robot	□							
8.	Jiang et al. (2021)	Tangible								□
9.	Mohadis et al. (2016)	Web App						□		
10.	Gomez-Carmona and Casado-Mansilla (2017)	Tangible								□
11.	Bootsman et al. (2019)	Tangible and Mobile App							□	
12.	Kronenberg et al. (2022)	Robot		□						
13.	Kim W. et al. (2019)	Robot							□	
14.	Bailly et al. (2016)	Actuators			□	□				

Mohadis et al. (2016) developed a low-fidelity web-based prototype to encourage physical activity among older office workers. They considered 23 persuasive principles as they relate to physical activity. These include reduction, tunneling, tailoring, personalization, self-monitoring, simulation, rehearsal, dialogue support, praise, rewards, reminders, suggestions, similarity, social role, credibility support expertise, real-world feel, third-party endorsements verifiability, social support/social learning, social comparison, normative influence, social facilitation, competition, and recognition. Reduction was targeted at making complex tasks simple to complete. Tunneling was driven by using the system to guide users while persuading them to change their behavior. Self-monitoring ensures that users can keep track of their behavior. Simulation covers demonstrating aspects of behaviors to interpret cause-and-effect relationships. Rehearsal provides an opportunity to continue to practice behavior toward change. In addition, the other persuasive principles (dialogue support praise, rewards, reminders, suggestions, similarity, social role, credibility support expertise, real-world feel, third-party endorsements verifiability, social support/social learning, social comparison, normative influence, social facilitation, competition, and recognition) were driven toward enhancing a change in the user's physical activity behaviors. The authors experimented with 10 participants and found that only two (2) persuasive principles were perceived positively. This includes dialogue support and credibility support.

Bootsman et al. (2019) explored wearable posture monitoring systems for nurses in workplaces. Nurses were considered to carry out repetitive bending throughout their work shifts. The system was designed to track their lower back posture. The system is connected to a mobile application that provides feedback on the different posture positions of users and tips for changing bad postures. The system was evaluated with six (6) nurses (aged between 20 and 65 years) for 4 days during work hours. Based on the intrinsic motivation inventory, the results show interest, perceived competence, usefulness, relatedness, and effort/importance scored more points. In addition, the results from the qualitative analysis show that participants appreciated the comfortability of the wearable system, though they were not in support of the frequency of beeps as it caused some distractions.

Haque et al. (2020) explored computer workstation movements similar to regular breaks. Unlike the regular break, computer users are encouraged to walk around and keep track of their physical activity level. The authors conducted an experiment with 220 office workers from the United Kingdom, Ireland, Finland, and Bangladesh for 4 weeks while evaluating their “IGO mHealth app.” The app monitors office workers' meal intake and work periods to send a 10-min interval walk-around reminder. The app tracks this movement while setting a target limit of 1,000 steps every 10 min. The app incorporates the leaderboard gaming element, encouraging competition through persuasion. The results from this study show a trend in weight loss, and a follow-up interview revealed three (3) persuasive principles that were perceived positively: (1) autonomy, (2) competence, and (3) relatedness. Autonomy shows how the app helped them achieve their set goals. Competence reflects how confident they were about their capability to use the app to perform different tasks. Relatedness shows how they were able to use the app to establish social connections.

Kronenberg et al. (2022) developed robotic arms that can be used to automatically adjust computer system screens. The robot detects the distance between the screen and the user's seating position. Then, the robot calculates the new screen orientation and adjusts to keep a healthy distance between the users and their computer screens. The authors conducted an experiment with 35 participants (25–68 years old) in their workspaces. The results of one-sample Wilcoxon Signed Rank Test show that participants could effectively complete the tasks and scenarios using this system at *(p* < 0.001), the screen did not move at the right pace when it moved (given that *p* = 0.189 was not significant that it moved at the right pace), the screen did not move at the appropriate moment (given that *p* = 0.904 was not significant that it moved at the appropriate moment), the screen was not well-adjusted to users' pose (given that *p* = 0.163 was not significant that it was well-adjusted to users pose), and the users felt distracted by the movement of the screen (given that *p* = 0.028 was not significant that users felt less distracted by the movement of the screen).

Kim M. T. et al. (2019) conducted experiments with a robot to support posture corrections during object lifting with 10 adults (30–34 years old). They considered five (5) different joints in the human body: (1) hips, (2) knees, (3) ankles, (4) shoulders, and (5) elbows. The results of their *t*-test analysis showed that the robot significantly lowered the overloading effect in all joints: shoulder (*p* < 0.001), elbow (*p* < 0.001), hip (*p* < 0.001), knee (*p* < 0.001), and ankle (*p* < 0.001). This implies that the robot can promote better posture practices in workplaces.

Bailly et al. (2016) developed a “*LivingDesktop”* that supports users to reduce reflection from the monitor screen. In addition, the system allows users to adjust the mouse and keyboard positions to improve ergonomics. The authors evaluated the system with 36 desktop users (22–40 years old). The results from this study show that users liked adjustable features because they fit their needs for video conferencing, tidying their workspace, and maintaining the right posture. On the other hand, some users criticized the system for its distractions in workspaces.

Jiang et al. (2021) developed a smart t-shirt wearable application for depression management in workplaces. They considered emotion regulation for depression management based on the movement of the shoulders and arms. The smart t-shirt changes resistance based on users' emotions. The fabric maintains a resistance of 180 kΩ while relaxed (positive emotion) and 400 kΩ when stretched (negative emotion). In view of this, they tested the smart t-shirt with six (6) healthcare workers for 5 days and found that the smart t-shirts regulated healthcare workers' emotions positively at work.

While most of these persuasive technologies have explored user interface design and user experience evaluation, we found other state-of-the-art practices employing machine learning techniques. Machine learning designs present more intelligent and data-oriented systems. This makes them more flexible to learn new patterns while users continue to interact with them. We present the extent to which machine learning has been tailored to enhance workplace practices in the next Section 2.3.

### 2.3 Machine learning and workplace practices

Machine learning can significantly impact the design of products for healthy workplaces. It interprets a wide range of data types, including sensor data, motion, eye movements, and human body movement. Machine learning models can be embedded into wearable devices, phones, and computers, enabling the detection of patterns in data and the optimization of communication with humans based on the diverse data they were trained on. For instance, facial recognition models, as supported in self-service photo booths (Kember, 2014), can detect specified height, width, and head position orientations (Chen et al., 2016).

Some significant research studies have delved into the application of machine learning in the realm of workplace practices. These studies have particularly focused on classifying healthy and active work styles (Rabbi et al., 2015) and automatic adjustments of chair and desk heights (Kronenberg and Kuflik, 2019). In their study, Kronenberg and Kuflik (2019) proposed a deep learning design for robotic arms that are capable of adjusting chair and desk heights based on body positions. Although the system was still in the implementation stage, initial results demonstrated the potential of embedding a camera in a robotic arm. This camera would interact with their proposed deep learning model.

Despite extensive research within this domain, limited study has been conducted on camera posture positions on the face, head, neck, and arms. While Min et al. (2015) explored body positions such as the back and spine using sensors, there is still a need to explore additional body positions captured by cameras. In a related study, Mudiyanselage et al. (2021) evaluated a workplace that involved lifting work-related materials using wearable sensors and various machine learning models (Decision Tree, Support Vector Machine, K-Nearest Neighbor, and Random Forest). The results indicated that the decision tree models outperformed others with a precision accuracy of 99.35%. Although these results were significant and focused on back body positions, there are still gaps within the context of computer workstations.

In another relevant study by Nath et al. (2018), significant work on lifting arm and wrist positions was considered using wearable sensors and the support vector machine (SVM) model. The study results demonstrated that SVM recognized over 80% of the risky positioning of the arm and wrist.

Hence, based on the persuasive and machine learning perspectives of workplace system design, different body positions are captured, and feedback is provided to support users. Nevertheless, there is a need to understand the extent to which research has supported making these technologies more accessible to diverse users. In the next section, we covered related work done with respect to making workplace posture technologies more accessible.

### 2.4 Accessibility technologies and healthy practices

Most accessibility technologies focus on providing feedback based on machine learning detection to address the needs of disabled individuals (Kulyukin and Gharpure, 2006). Brik et al. (2021) developed an IoT-machine learning system designed to detect the thermal comfort of a room for disabled persons, offering feedback on the room's thermal condition. The machine learning system was trained on artificial neural networks (ANNs). The performance of ANNs was compared with other algorithms such as logistic regression classifiers (LRC), decision tree classifiers (DTC), and gaussian naïve bayes classifiers (NBC). ANN performed better, achieving 94% accuracy compared with the other algorithms.

In a related study, Ahmetovic et al. (2019) investigated navigation-based assistive technologies for the blind and visually impaired. They identified rotation errors and utilized a multi-layer perceptron machine learning model to correct rotation angles, providing positive feedback. The multi-layer perceptron achieved lower rotation errors (18.8° on average) when tested with 11 blind and visually impaired individuals in real-world settings.

Overall, we found that though related studies have explored healthy practices in workplace settings based on different persuasive technologies ranging from mobile to tangible, little work has covered real-time posture detection for important areas of the body such as the back, neck, hands, and head. These parts of the body have been associated with a lot of repetitive workplace stress injuries based on bad postures (Anderson and Oakman, 2016; Catanzarite et al., 2018; Krajnak, 2018). The study by Min et al. (2015) and Mudiyanselage et al. (2021) presents closely related concepts. Though these studies explored parts of the body such as the back, spine, arm, and wrists, they used sensors, which might not be comfortable for users of systems. Considering that laptop cameras can detect these parts of the body in an unobstructive way, we explored this in our current study.

## 3 Materials and methods

We outline the materials and methods employed in the study. This aligns with the overarching goal of our research to investigate how individuals can become aware of their unhealthy posture practices in workplaces (both while sitting and standing) and the main research question (RQ: Can persuasive computers be designed to detect unhealthy posture practices in workplaces?). We provide details on the experimental materials used for developing deep learning models, specifically convolutional neural networks and Yolo-V3.

### 3.1 Data collection and preprocessing

**Figure 4:**
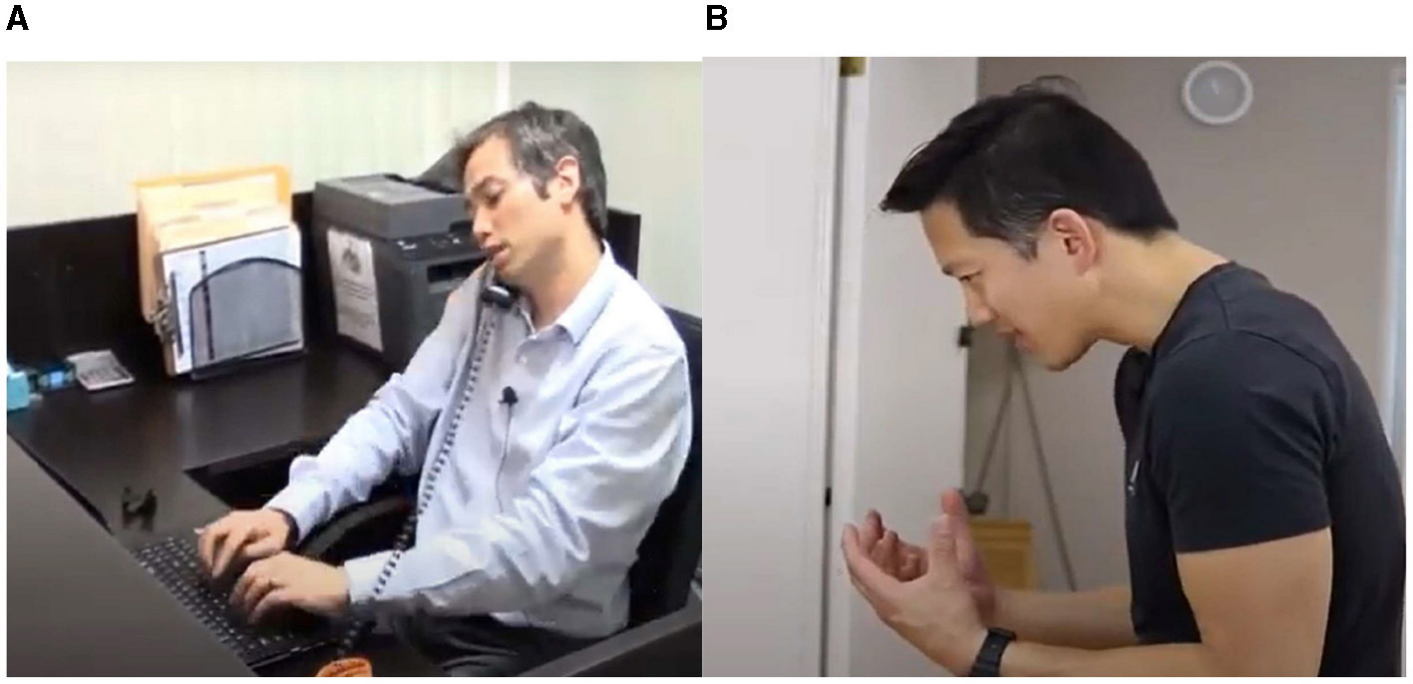
Samples of bad practices. **(A)** Reproduced from “Center for Musculoskeletal Function: Workspace Ergonomics and MicroBreak Exercises,” YouTube, uploaded by “Dr. Daniel Yinh DC MS,” 10 Apr 2017, https://www.youtube.com/watch?v=HS2KrPmKySc, Permissions: YouTube Terms of Service. **(B)** Reproduced from “Correct Ergonomic Workstation Set-up | Daily Rehab #23 | Feat. Tim Keeley | No.112 | Physio REHAB,” YouTube, uploaded by “Physio REHAB,” 13 December 2017, https://www.youtube.com/watch?v=FgW-9_28N8E&t=314s, Permissions: YouTube Terms of Service.

**Figure 5:**
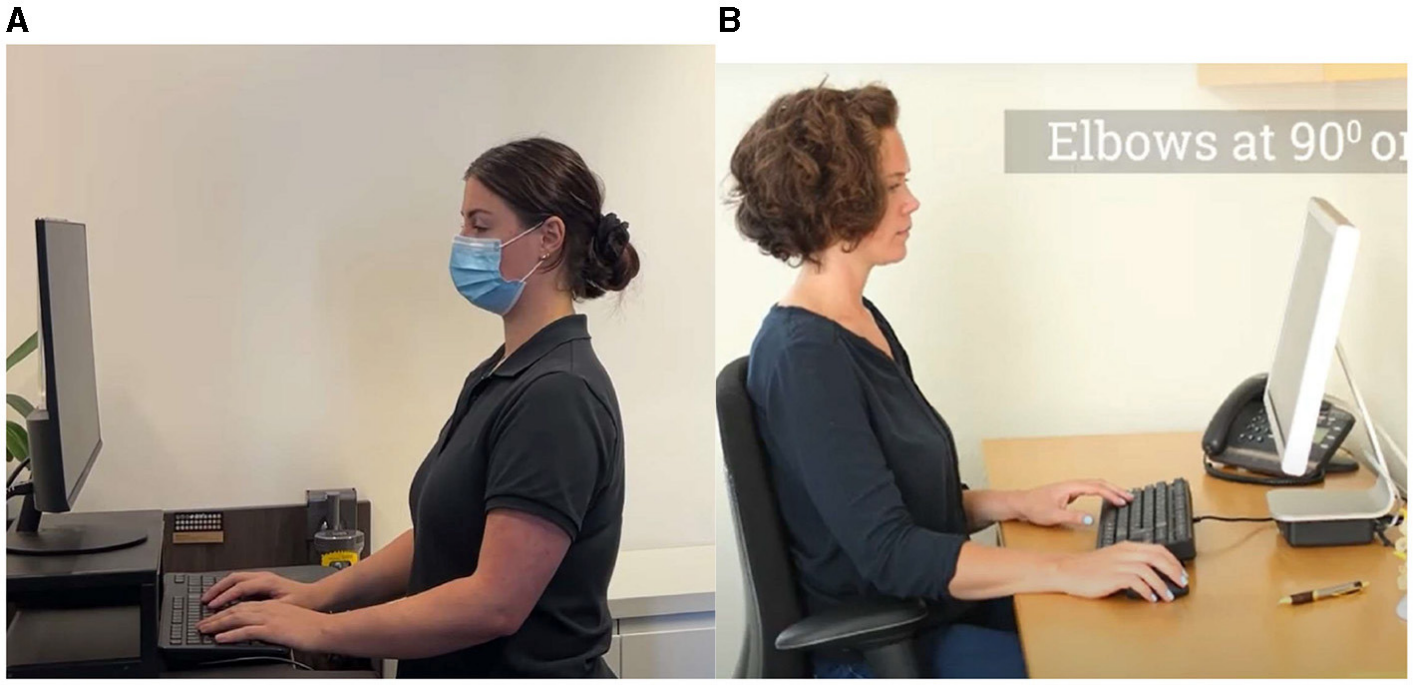
Samples of the good practices. **(A)** Reproduced from “Working from home—how to set up your laptop (correctly!) | Tim Keeley | Physio REHAB,” YouTube, uploaded by “Physio REHAB,” 19 March 2020, https://www.youtube.com/watch?v=6GlkoFnZpFk, Permissions: YouTube Terms of Service. **(B)** Reproduced from “How to set up workstation at home,” YouTube, uploaded by “Sundial Clinics,” 12 April 2021, https://www.youtube.com/watch?v=wN-Ww1sCWNY, Permissions: YouTube Terms of Service.

We conducted data collection in three phases (phase 1, phase 2, and phase 3). In the first phase, we gathered data by extracting Creative Commons image datasets from YouTube using the search terms ({bad} OR {good} AND {ergonomic posture}). Utilizing the Snip and Sketch tools, we extracted key frames depicting instances of both good and bad ergonomics. In total, we amassed 269 image datasets, comprising 157 examples of bad practices and 112 examples of good practices. The datasets from this initial phase were utilized for the pilot study, which aimed to assess the feasibility of employing machine learning for the detection of posture practices. Figures 4, 5 provide a cross-section of the datasets collected from YouTube.

In addition, we gathered more image datasets from Pexels using the Snip and Sketch tools. Pexels offers royalty-free images that match both the good and bad workplace practices of computer users. Utilizing related search terms such as “people AND {using the computer}” OR “{looking head straight}” OR “{sitting in the office},” we extracted key frames, resulting in 618 instances of bad practices and 90 instances of good practices. These datasets were combined with those from Phase 1 to conduct the main study for YOLO-V3.

**Figure 6:**
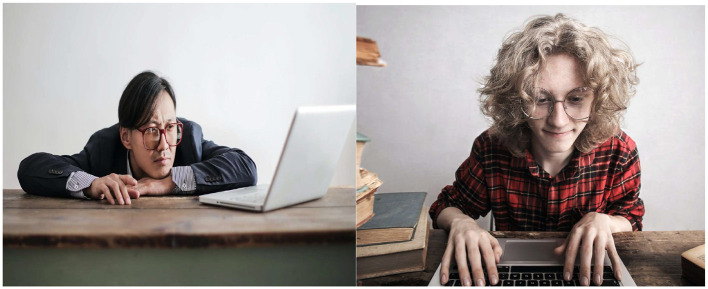
Samples of bad posture. Reproduced from Pexels.

**Figure 7:**
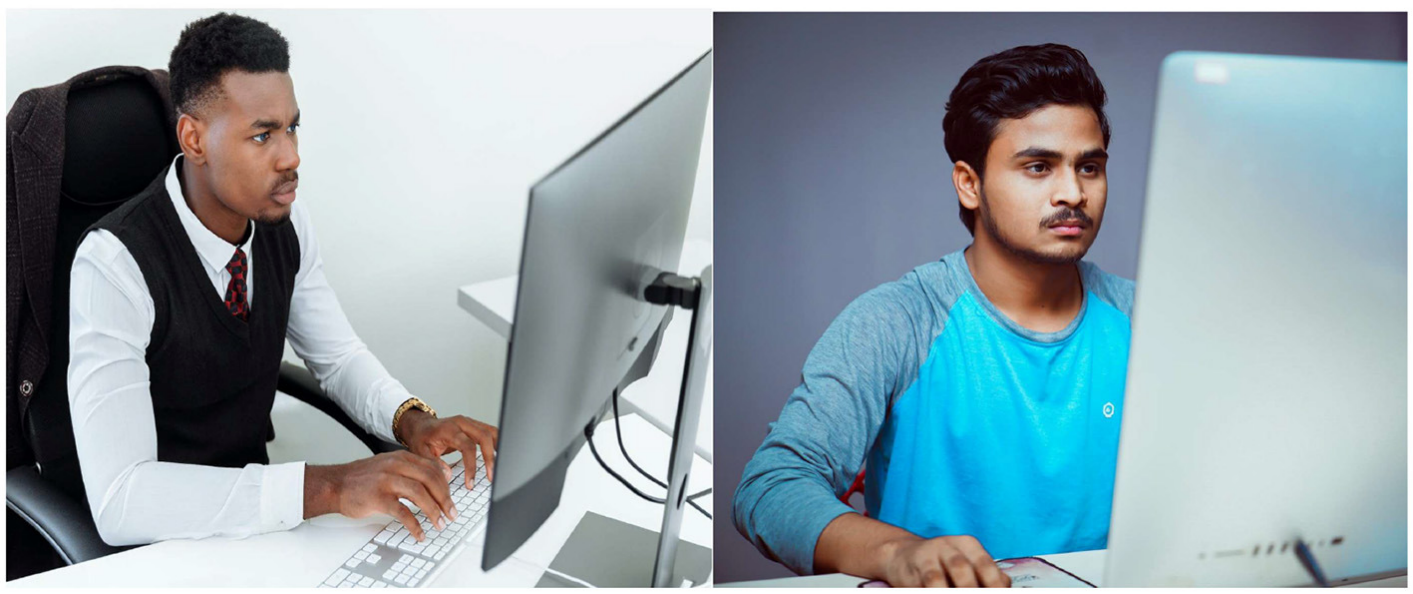
Samples of good posture. Reproduced from Pexels.

Recognizing the limitations of convolutional neural networks (CNN) with small datasets (Han et al., 2018), we addressed this concern in Phase 3 by collecting additional datasets. To enhance the dataset, we collected both zoomed-in and zoomed-out resolution images from Pexels. Research has shown that zooming, as one of the techniques of data augmentation, increases the number of datasets (Shorten and Khoshgoftaar, 2019). Figures 6, 7 offer a cross-section of the datasets collected from Pexels.

**Figure 8:**
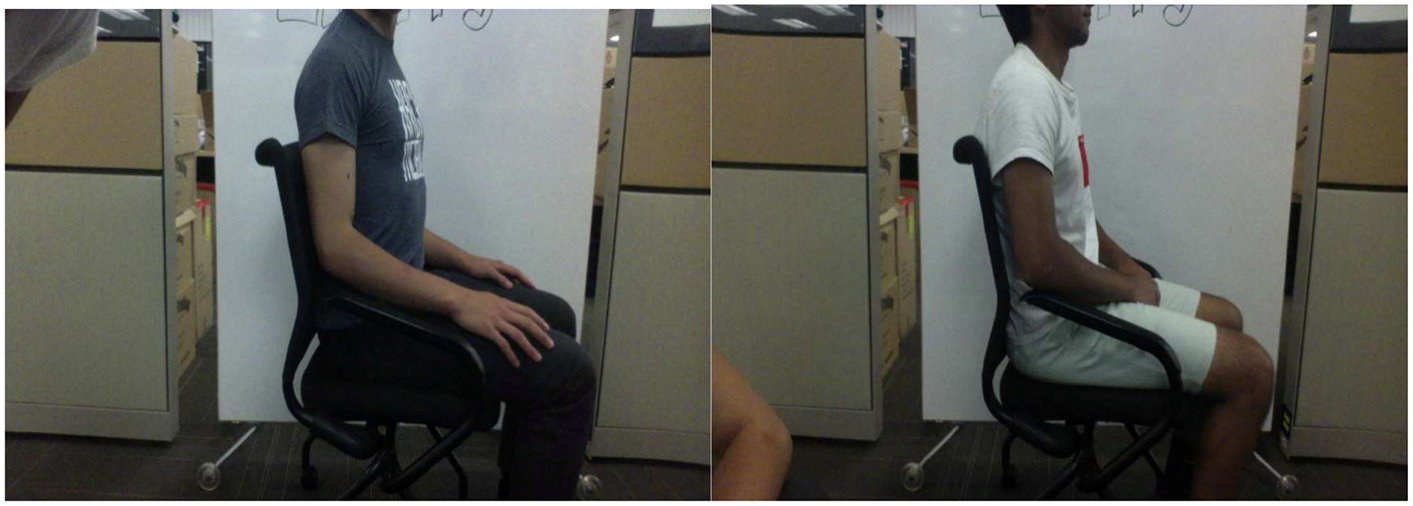
Samples of the good practices. Reproduced from Kaggle.

For the Phase 3 data collection task, we explored the posture dataset available on Kaggle. Kaggle, known for its extensive repository of public datasets for machine learning (Tauchert et al., 2020), provided a valuable resource. We added 311 images depicting good practices to the datasets from Phases 1 and 2. The combined datasets from this phase were used to conduct the main study experiment for convolutional neural networks (CNN). Figure 8 showcases a cross-section of sample images collected from Kaggle9. Though Kaggle had a couple of images for bad postures, we considered using the good ones to balance our datasets (we initially had more bad postures compared with good postures).

Additionally, we defined the two classes as “comfortable” and “uncomfortable.” All the image datasets depicting good practices were assigned to the “comfortable” class, while those depicting bad practices were assigned to the “uncomfortable” class. Table 3 offers a summary of all the datasets collected for the study. We employed static image datasets as they are applicable to existing real-time detection studies (Huang et al., 2019; Lu et al., 2019), and a video is a sequence of moving images in frames (Lienhart et al., 1997; Perazzi et al., 2017). Hence, the computer vision library provides functionality to help capture this image frame per second and parse them to the machine learning model to quickly predict the class in real time.

**Table 3:** Summary of datasets distribution by source.

**S/N**	**Source**	**Comfortable**	**Uncomfortable**
1.	YouTube	112	157
2.	Pexels	90	618
3.	Kaggle	311	-
Total		513	775

### 3.2 Study description

We covered two significant steps, namely, the pilot and main studies. We explored the feasibility of designing with a few datasets in a pilot study. We present this pilot study to guide the research community on the impact of dataset size in this area. In the main study, we extended the number of datasets to show improvements in the accuracy of models. The datasets collected from YouTube during Phase 1 data collection were pre-processed and used to train the two models for the pilot study (CNN-pilot and Yolo-V3-pilot). We evaluated their performance through loss graphs and in real-time (mean average precision). The mean average precision is a metric for evaluating the accuracy of object detection, especially in real time (Padilla et al., 2021). Furthermore, we combined datasets from YouTube and Pexels to train the YOLO-V3-main model. Additionally, we combined datasets from YouTube, Pexels, and Kaggle to train the CNN-main model. Both the YOLO-V3-main and CNN-main models were developed for the main study.

#### 3.2.1 Pilot study

We conducted two experiments for the pilot study. The first experiment involved the development of the Yolo-V3 model (Yolo-V3-pilot). We performed an automatic data annotation task^1^ on the entire datasets collected from YouTube. Subsequently, we trained our datasets on the Yolo-V3 model implementation of keras-yolo3^2^ on the CPU and we tested this implementation on Google Colab. The second experiment was implemented on the CNN model of Abhishekjl.^3^ Our selection of Abhishekjl's framework was based on its relevance in the application of the cv2 python library which is applicable in the recent study by Singh and Agarwal (2022). In addition, the keras-yolo3 implementation has been recently applied to the current state-of-the-art pedestrian detection system by Jin et al. (2021) and other systems (Chen and Yeo, 2019; Silva and Jung, 2021). Hence, datasets collected from YouTube were trained on the CNN model (CNN-pilot). The CNN-pilot model was trained and tested on Google Colab.

#### 3.2.2 Main study

We conducted two experiments for the main study. In the first experiment, we combined datasets from YouTube and Pexels (from phases 1 and 2 of data collection). We performed automatic data annotation exclusively for datasets from Pexels. The annotation data were then added to pre-existing annotations from the pilot study to train a new Yolo-V3 model (Yolo-V3-main) for the main study, utilizing CPU resources. In the second experiment, we combined datasets from YouTube, Pexels, and Kaggle (from phases 1–3) and trained them using Google Colab on the CNN model (CNN-main). Like the pilot study, both Yolo-V3 and CNN models were implemented based on the architectures of Keras-Yolo3 and Abhishekjl. In addition, we tested Yolo-V3-main and CNN-main in Google Colab.

### 3.3 Overview of the CNN model

**Figure 9:**
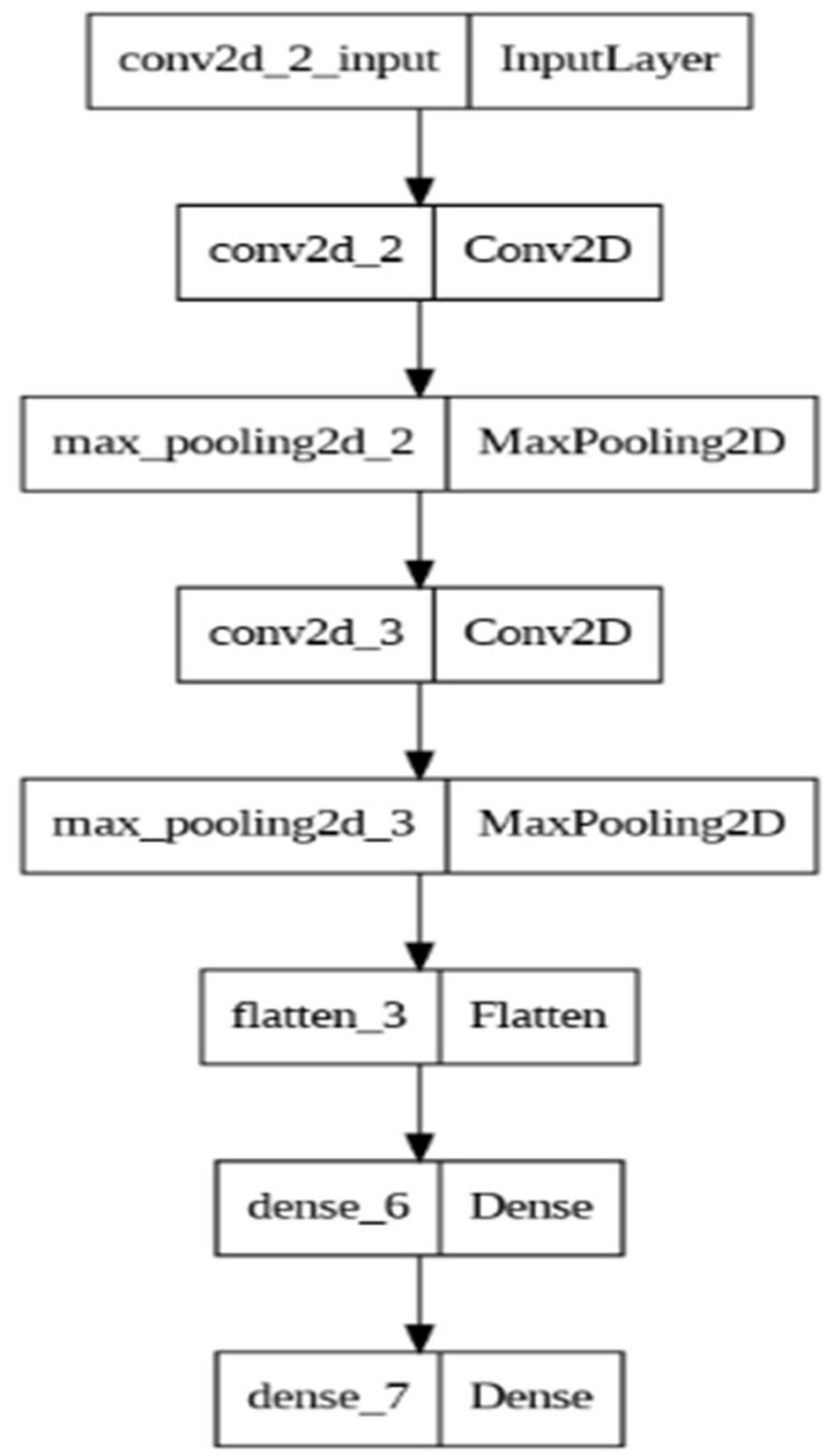
CNN model architecture.

The CNN model (Figure 9) consists of 2 convolutional 2D layers, 2 max_pooling 2D layers, one flatten, and twi dense layers. Furthermore, the hyperparameters for the model include 3 activation functions (rectified linear unit, RELU) for the convolutional 2D layers and one of the dense layers, one sigmoid activation function added to the last dense layer, Adam optimizer, a learning rate of 1e-3, a batch size of 5, and 10 epochs. The loss of the CNN-pilot model was set to binary_crossentropy. The convolutional 2D layers combine the 2D input after filtering, computing the weights, and adding a bias term (Li et al., 2019). The max_pooling2d layers reduce the input dimensions, leading to a reduction in outputs (Keras^4^). The flatten layer combines all the layers into a flattened 2-D array that fits into the neural network classifier (Christa et al., 2021). The dense layers are regular, deeply connected neural network layers that are used to return outputs from the model (Keras^5^). We employed the rectified linear unit (RELU) activation function as it is one of the most widely used functions because of its improved performance (Dubey et al., 2022). The sigmoid function was selected because it is suitable for binary classification tasks (Keras^6^) as we employed in our study. We employed the Adam optimizer because it is memory efficient and requires limited processing resources (Ogundokun et al., 2022). We set the learning rate of 1 e-3 and batch size 5 as we considered the sensitivity of CNN models to small datasets (Brigato and Iocchi, 2021).

### 3.4 Overview of the Yolo-V3 model

**Figure 10:**
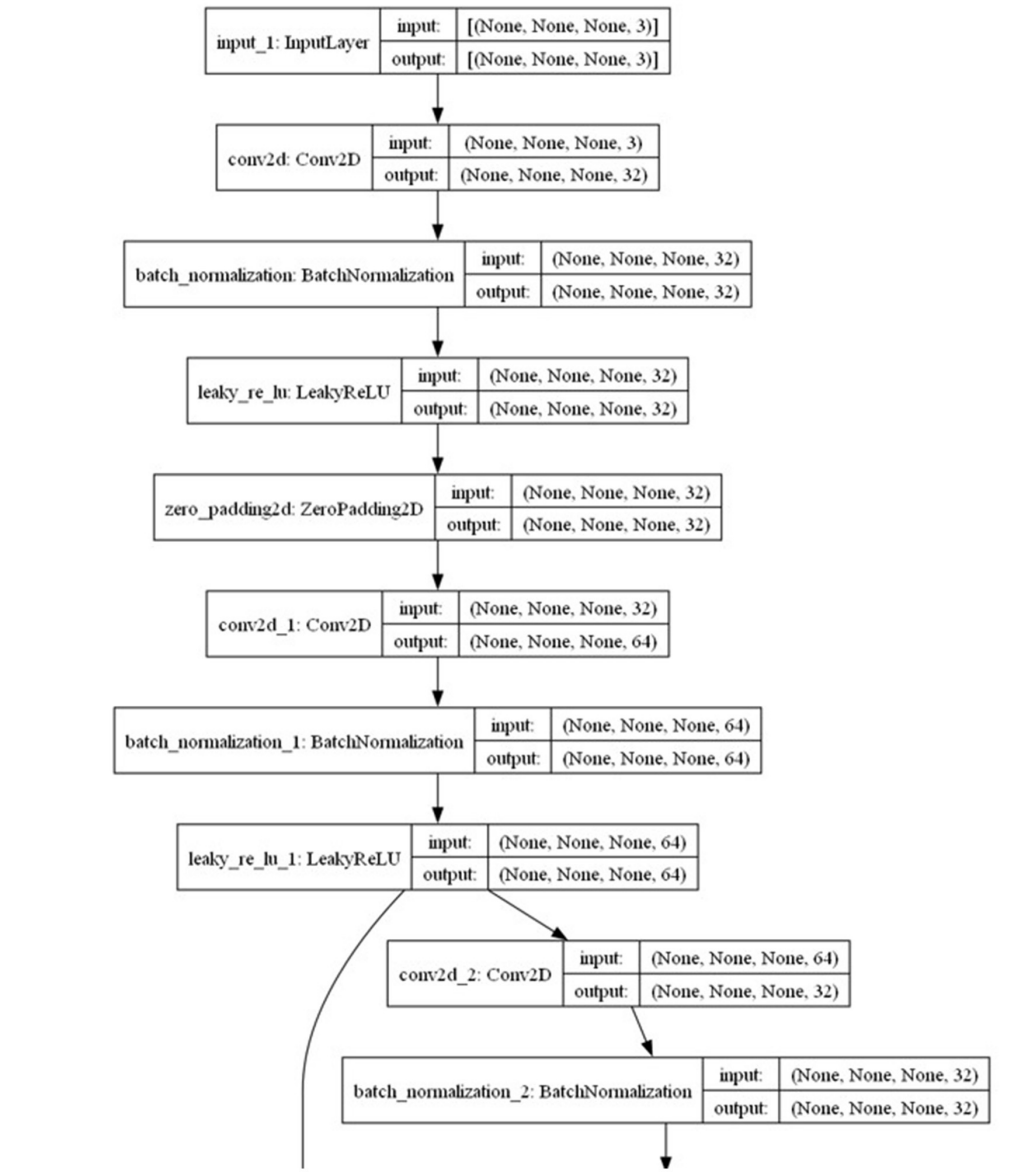
Cross-section of the YOLO-V3 model architecture (full architecture is available at Appendix A1).

The Yolo-V3 model (Figure 10) consists of 74 convolutional 2D layers, 71 batch normalization layers, 70 leaky rectified linear unit (RELU) activation layers, two UpSampling2D layers, and one ZeroPadding2D layer. We set the hyperparameters for the model as follows: Adam optimizer, learning rate of 1e-4, and batch size of 16. We consider Adam Optimizer to be appropriate as it is memory efficient and requires limited processing resources (Ogundokun et al., 2022). In addition, we considered a reduced learning rate and batch size because of the number of datasets we have. This will help the model learn efficiently. Unlike CNN, YOLO-V3 yielded more annotated datasets with different dimensions. This is typical with YOLO-V3 data annotations (Diwate et al., 2022). Furthermore, we varied the number of epochs for both the pilot and main studies. We used four epochs for the pilot study (Section 4.12) and a maximum of 40 epochs for the main study (Section 4.2.2). We used the default loss function (binary_crossentropy) for the YOLO model. The convolutional 2D layers combine the 2D input after filtering, computing the weights, and adding a bias term (Li et al., 2019). The batch normalization layer normalizes inputs to ensure that they fit the model as their weights continue to change with each batch that the model processes (Arani et al., 2022; Keras^7^). The leaky RELU activation layer is a leaky version of a rectified linear unit activation layer (Keras^8^). It introduces non-linearity among the outputs between layers of a neural network (Xu et al., 2020). The UpSampling2D layer is used to repeat the dimensions of the input to improve its quality (Liu et al., 2022; Keras^9^). The ZeroPadding2D layer adds extra rows and columns of zeros around images to preserve their aspect ratio while being processed by the model (Dang et al., 2020; Keras^10^).

## 4 Results

In this section, we present our findings from the pilot and main studies. This section covers reports from our experiments with Yolo-V3 and CNN models using datasets collected from YouTube, Pexels, and Kaggle.

### 4.1 The pilot study

To visualize the feasibility of the study, we developed two models for detecting workplace practices in real time: CNN and Yolo-V3. We chose these models based on their proven capabilities for supporting real-time object detection in previous research (Tan et al., 2021; Alsanad et al., 2022). For the CNN model, we divided the datasets into 75% training and 25% validation datasets (refer to Table 4). We used 75% training to 25% validation set split for the CNN model considering how similar tasks employed this ratio (Azimjonov and Özmen, 2021; Bavankumar et al., 2021; Akter et al., 2022). Programmatically, we split the datasets into 90% training and 10% validation datasets for the Yolo-V3 model. The reason for the difference in this split ratio was based on previous studies employing similar ratios, especially for Yolo models (Akut, 2019; Setyadi et al., 2023; Wong et al., 2023).

**Table 4:** Summary of dataset distribution for the pilot study.

**S/N**	**Model**	**Comfortable**		**Uncomfortable**		**Total**	
		**Training**	**Validation**	**Training**	**Validation**	**Training**	**Validation**
1.	CNN	84	28	118	39	202	67
2.	Yolo-V3	101	11	141	16	242	27
Total		185	39	259	55	444	94

#### 4.1.1 CNN pilot study posture detection

**Figure 11:**
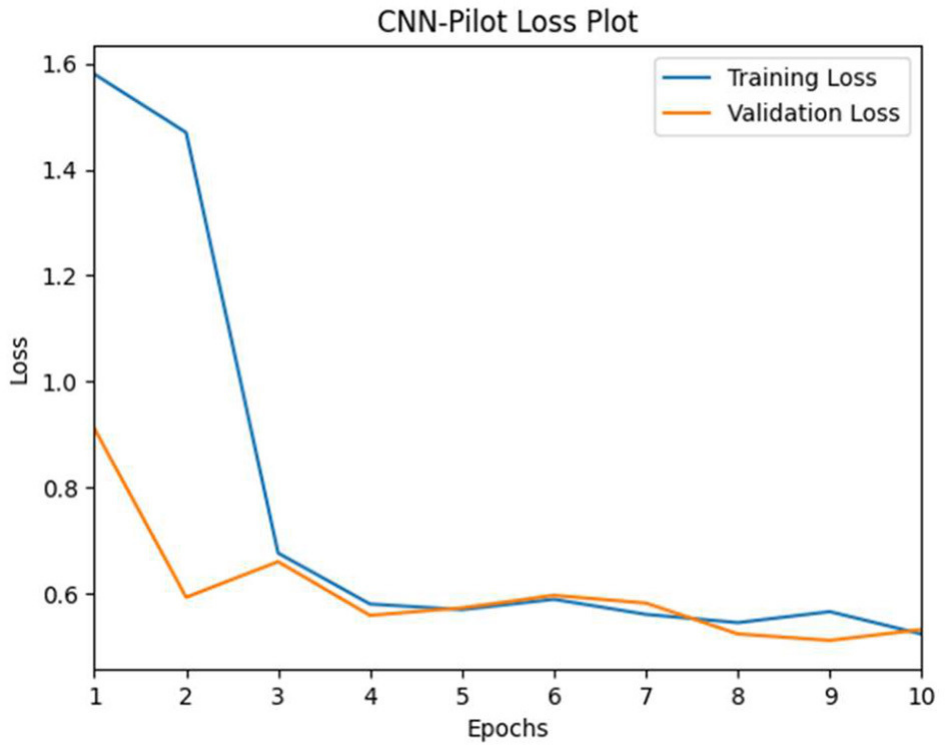
CNN-pilot model's training vs. validation loss.

We trained the CNN-pilot model for 10 epochs, employing hyperparameter tuning variables such as the stochastic gradient descent optimizer with a learning rate of 1e-3. The results of our CNN training indicate a significant decrease in both training and validation loss values, approaching the 10th epoch (see Figure 11). The validation loss was minimal at epoch 10 compared with the training loss, suggesting a slight underfitting of the model.

**Figure 12:**
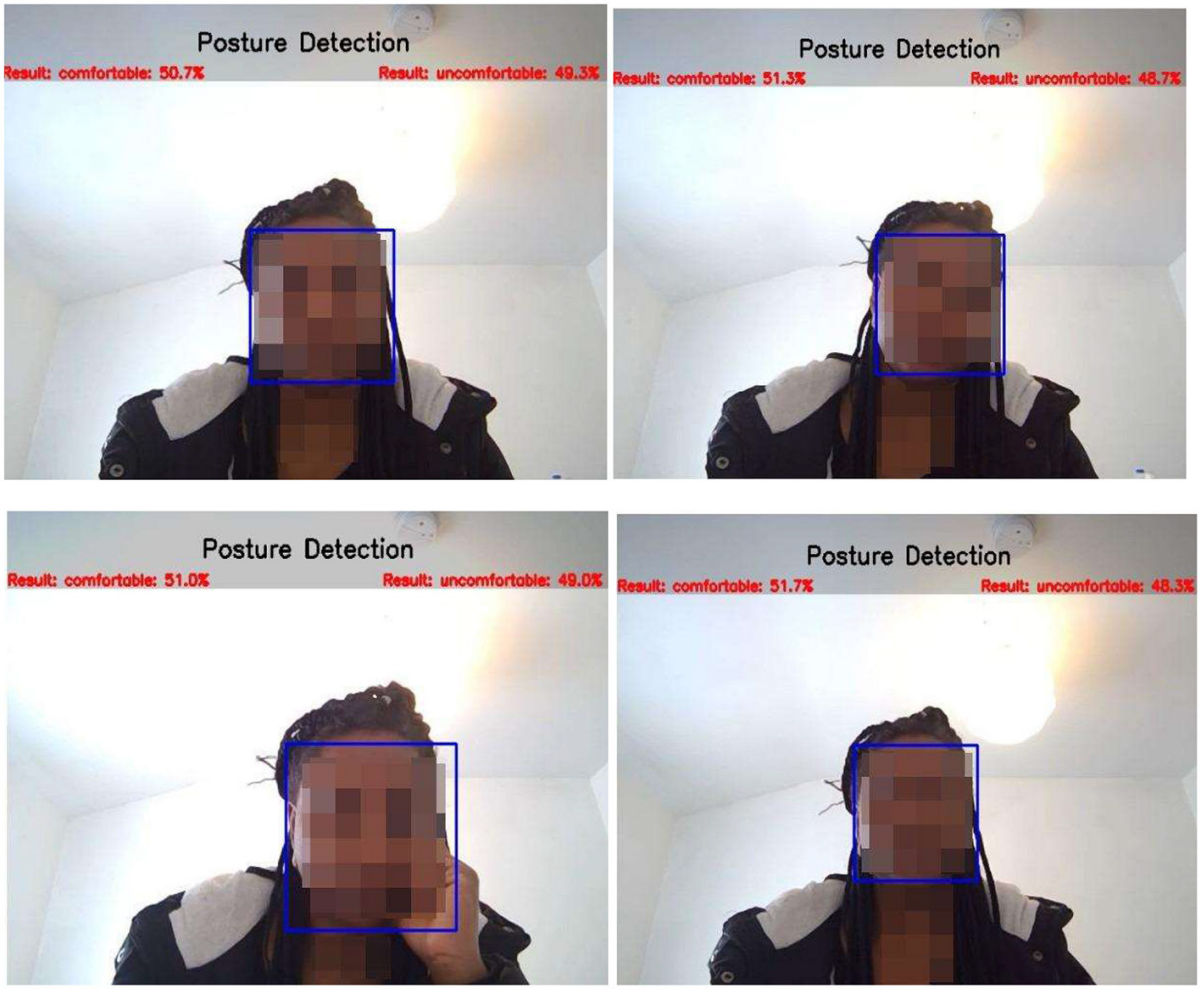
CNN-pilot model's detection of posture.

We deployed the model in real-time using the computer vision Python library. Running the model on six real-time test instances, it achieved a mean average precision of 52%. In most instances, better precision values were observed for “comfortable” compared with “uncomfortable” (see Figure 12).

#### 4.1.2 Yolo-V3 pilot study posture detection

**Figure 13:**
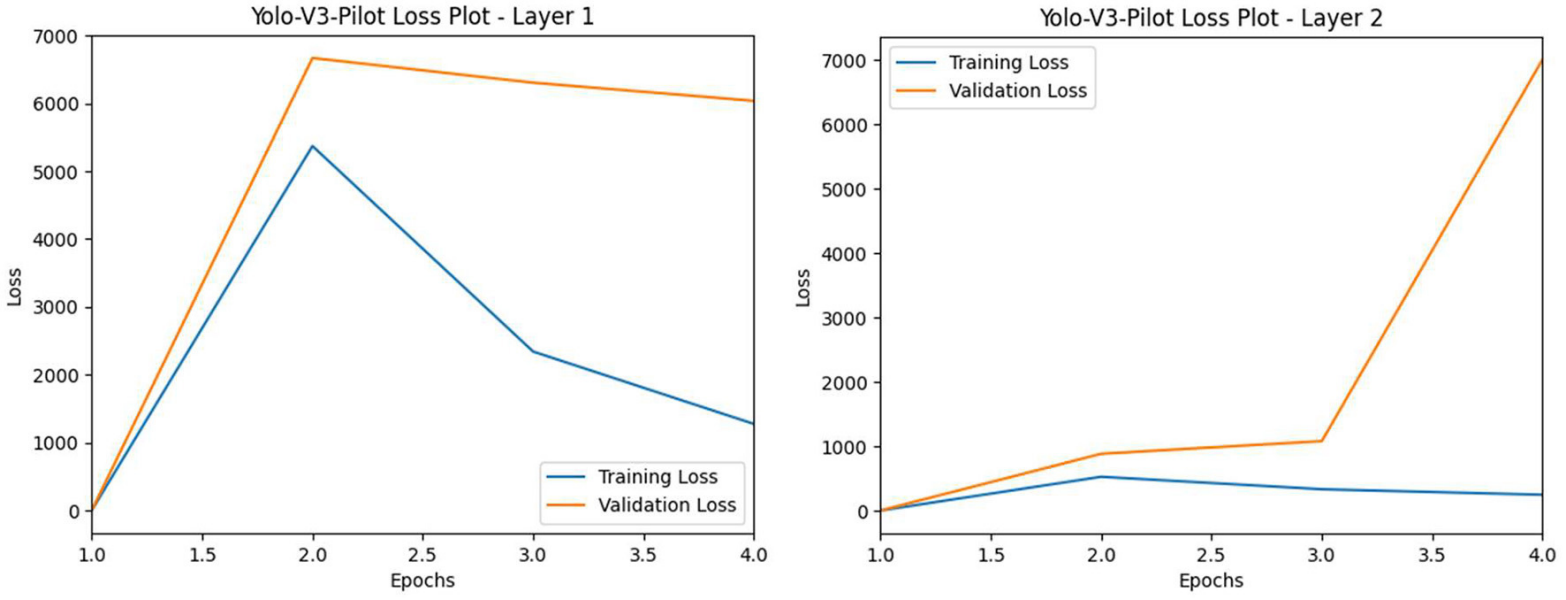
L-R: Yolo-V3-pilot model's training vs. validation loss (L: Layer 1 and R: Layer 2).

The Yolo-V3-pilot model was trained with two layers, employing a strategy of frozen layers to stabilize the loss and unfrozen layers to further reduce the loss, over four epochs. These layers were configured to train with hyper-tuning parameters, including the Adam optimizer with a learning rate of 1e-4 and a batch size of 16. The results of our YOLO-V3 layers 1 and 2 training reveal a decrease in the training loss toward epoch 4 compared with the validation loss (refer to Figure 13). However, it is typical for YOLO-V3 to return a high level of loss values below epoch 10 (Li et al., 2020).

**Figure 14:**
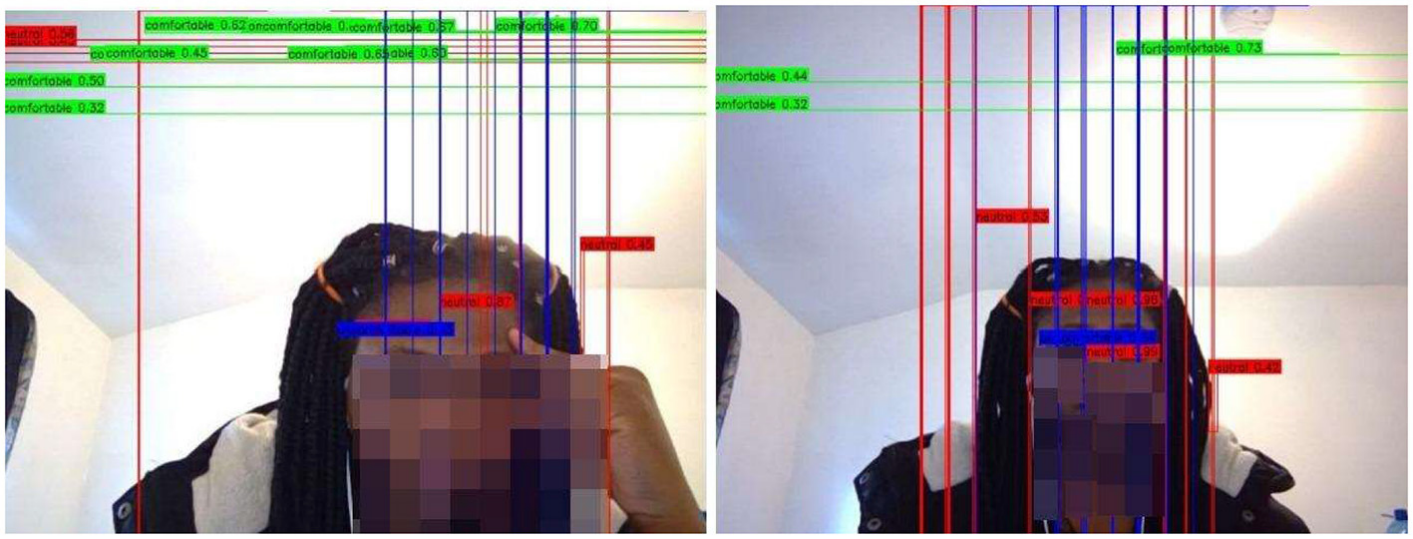
L-R: Yolo-V3-pilot model's posture detection: 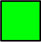 comfortable; 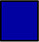 uncomfortable; 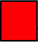 neutral. L: showing areas of discomfort around the eyes and where the hand intercepts the eyes. R: showing discomfort from the eye to the neck regions.

**Figure 15:**
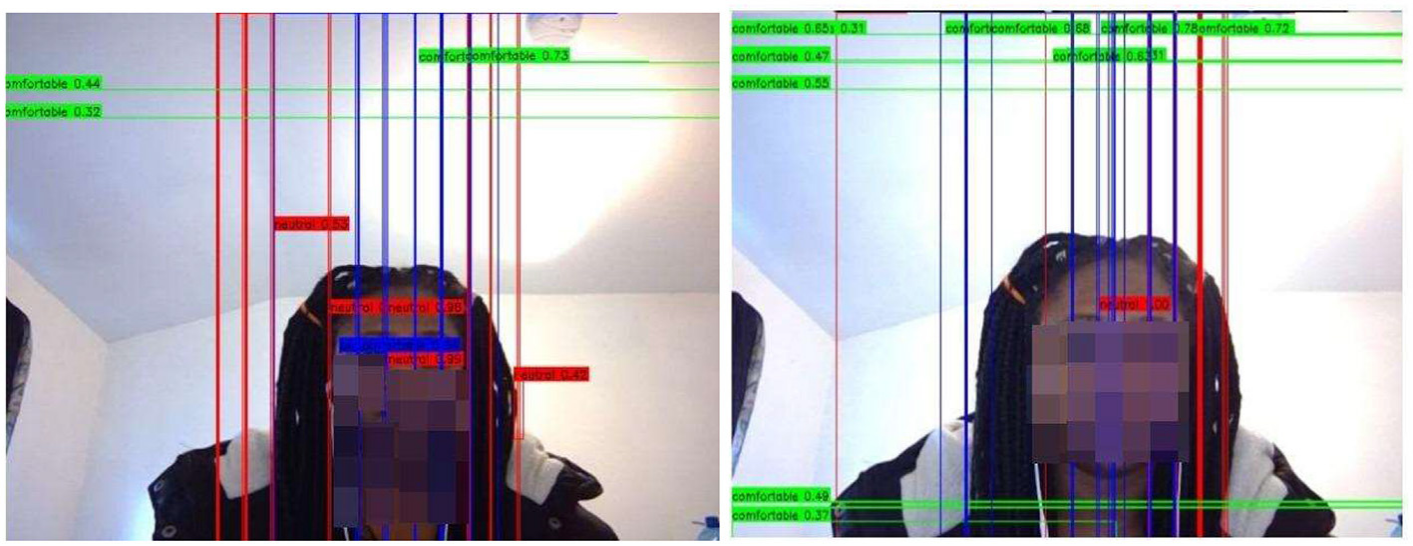
L-R: Yolo-V3-pilot model's posture detection: 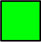 comfortable; 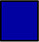 uncomfortable; 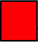 neutral. L: showing areas of discomfort around the eyes, neck, and back regions. R: showing discomfort from the eye to the neck regions.

We deployed the Yolo-V3-pilot model in real time for the classes “comfortable” and “uncomfortable.” For exceptional cases, we included a “neutral” class. This addition allows Yolo-V3 to handle instances where the detections do not match the expected classes. Figures 14, 15 showcase instances where the Yolo-V3-pilot model segmented areas of comfort compared with discomfort. In other cases, the model returned “neutral” while one of the researchers tested it in real time using the computer vision Python library. The model achieved a mean average precision of 64% across six real-time test instances.

From the results of both models (CNN-pilot and Yolo-V3-pilot), the Yolo-V3-pilot model's boxes extended beyond the face, capturing other significant areas of comfort or discomfort such as the eyes, neck, and back (see Figures 14, 15).

### 4.2 The main study

To enhance the performance of both models (CNN-main and Yolo-V3-main) in the main study, we trained these models on additional datasets collected from Pexels and Kaggle. For the Yolo-V3-main model, we combined YouTube datasets with those from Pexels, while the CNN-main model was trained on a combination of datasets from YouTube, Pexels, and Kaggle. In the case of the CNN-main model, we split the datasets into 75% training and 25% validation sets (refer to Table 5). We maintained the 90% training and 10% validation set split for the Yolo-V3-main model.

**Table 5:** Summary of dataset distribution for the main study.

**S/N**	**Model**	**Comfortable**		**Uncomfortable**		**Total**	
		**Training**	**Validation**	**Training**	**Validation**	**Training**	**Validation**
1.	CNN	384	129	581	194	965	323
2	Yolo-V3	182	20	698	77	880	97
**Total**		**566**	**149**	**1,279**	**271**	**1,845**	**420**

#### 4.2.1 CNN main study posture detection

**Figure 16:**
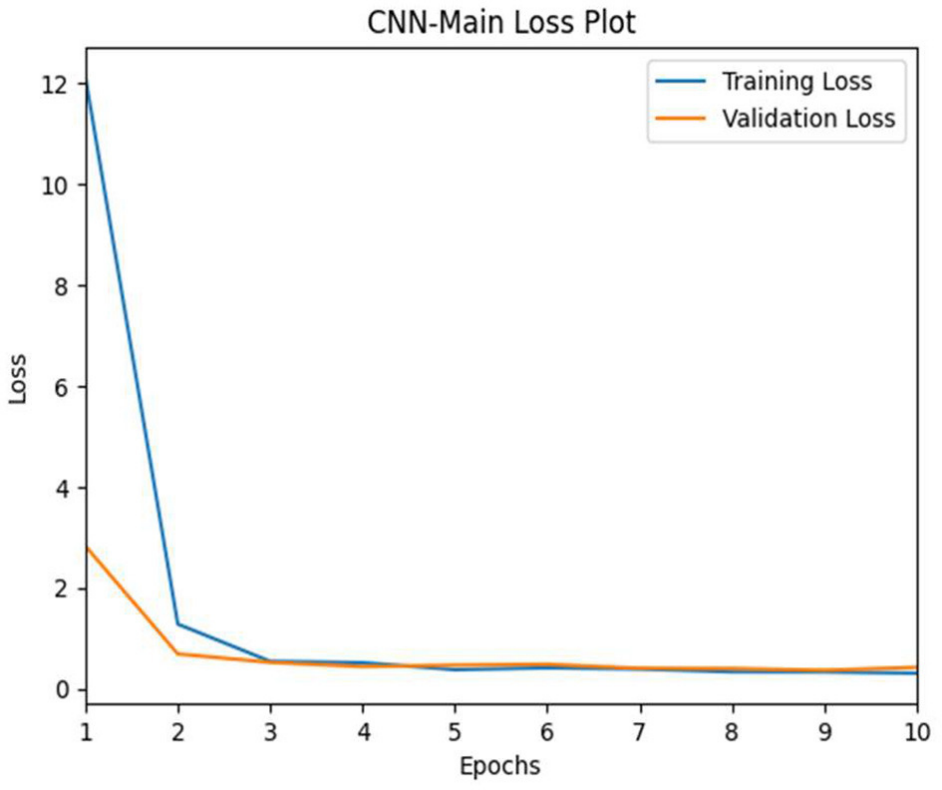
CNN-main model's training vs. validation loss.

We maintained the hyper-tuning parameters from the pilot study for CNN, and the model was trained for 10 epochs. The results of our CNN training indicate a significant decrease in both training and validation loss values, approaching the 10th epoch (see Figure 16). The training loss was minimal at epoch 10 compared with the validation loss, indicating better convergence of the training and validation losses compared with those reported earlier in the pilot study (see Figure 11).

**Figure 17:**
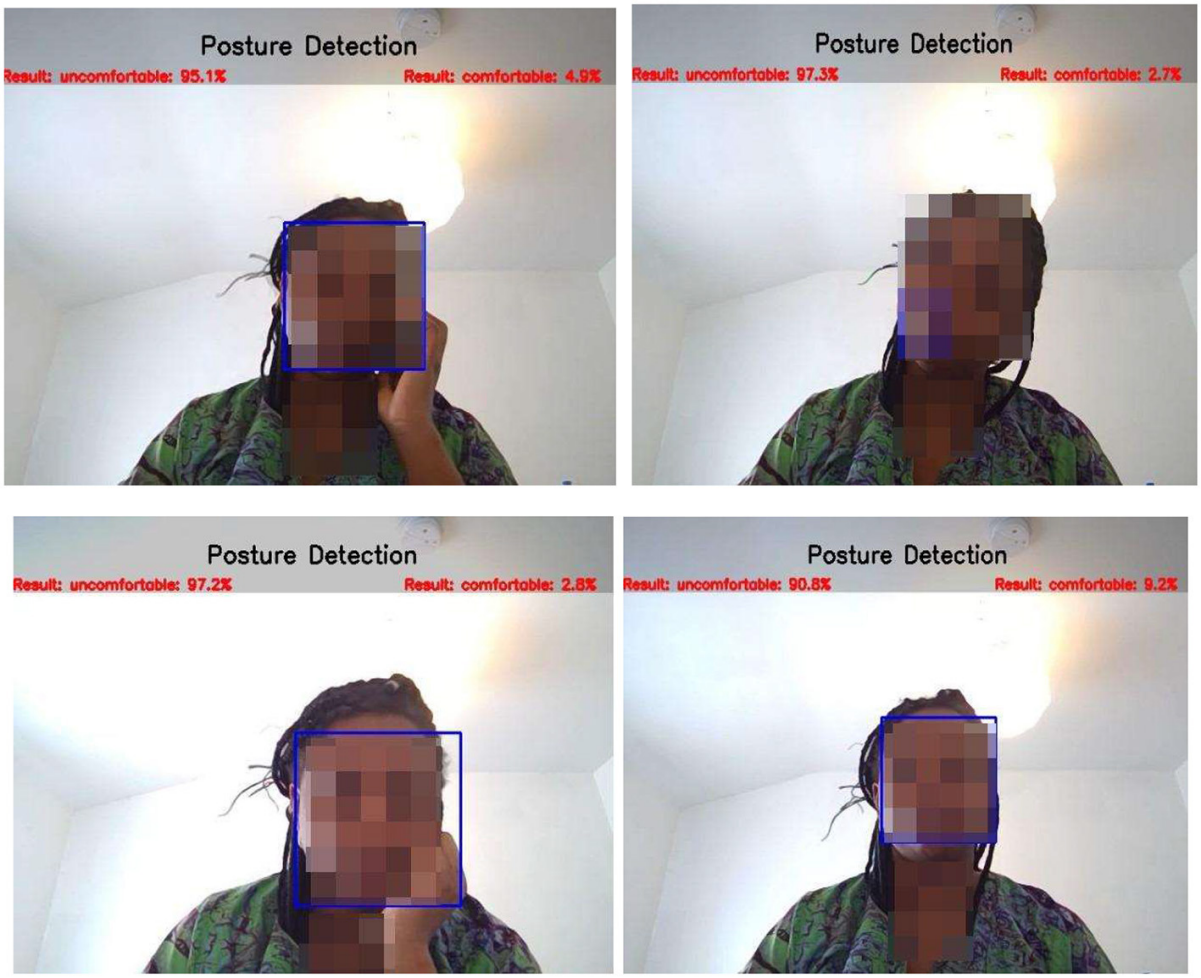
CNN-main model's posture detection.

In real time, the CNN-main model predicts uncomfortable classes better (Figure 17: 89.6, 98.7, 93.5, and 93.0%). The CNN-main model attained a mean average precision of 91% on 19 real-time test data points.

#### 4.2.2 Yolo-V3 main study posture detection

**Figure 18:**
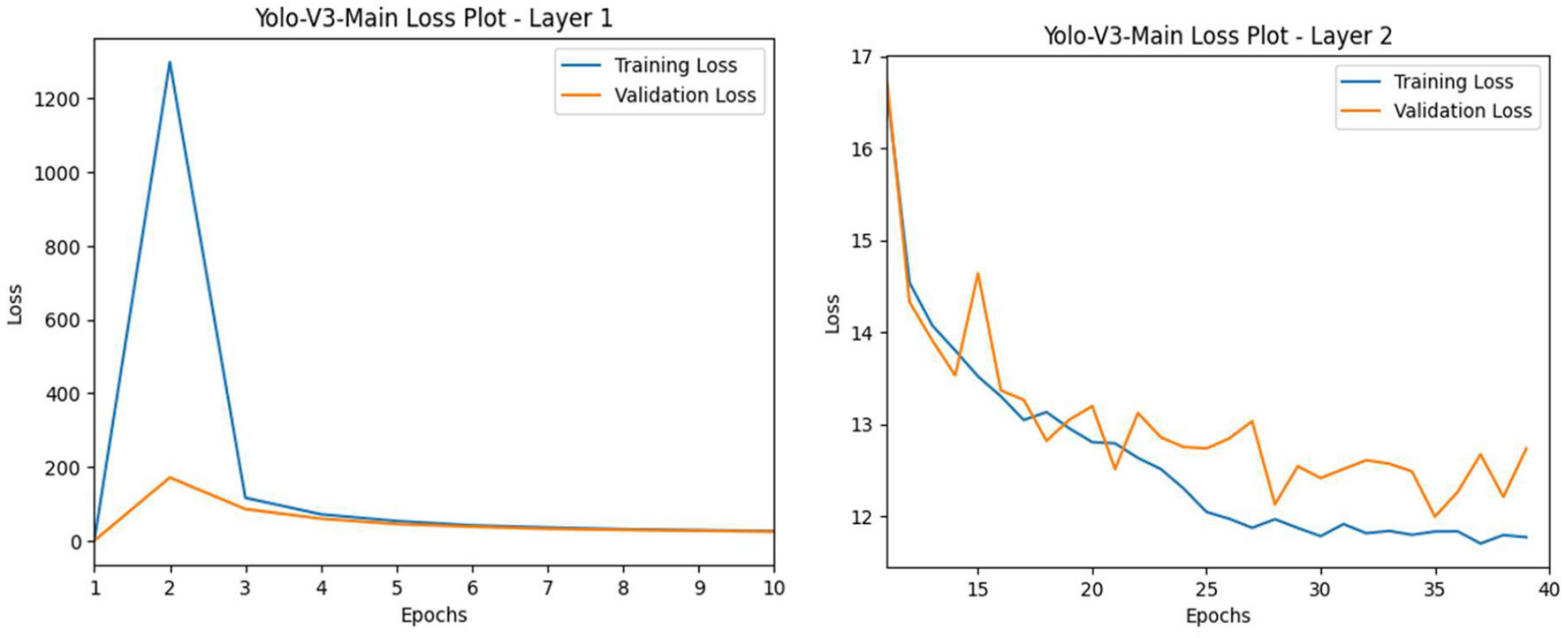
L-R: Yolo-V3-main model's training vs. validation loss (L: Layer 1 and R: Layer 2).

Like the pilot study, the Yolo-V3-main model was trained with two layers, incorporating frozen layers for a stable loss and unfrozen layers to further reduce the loss. The first layer was set to train for 10 epochs, and the second layer started at the 11th epoch (continuing from the first layer) and concluded at the 39th epoch. These layers were trained with hyper-tuning parameters, including the Adam optimizer with a learning rate of 1e-4 and a batch size of 16. The results for both layers 1 and 2 of the Yolo-V3-main model show that the training and validation loss curves converged at epoch 10 for the first layer and diverged slightly upward at epoch 39 for the second layer (see Figure 18). This implies slight overfitting of our Yolo-V3-main model.

**Figure 19:**
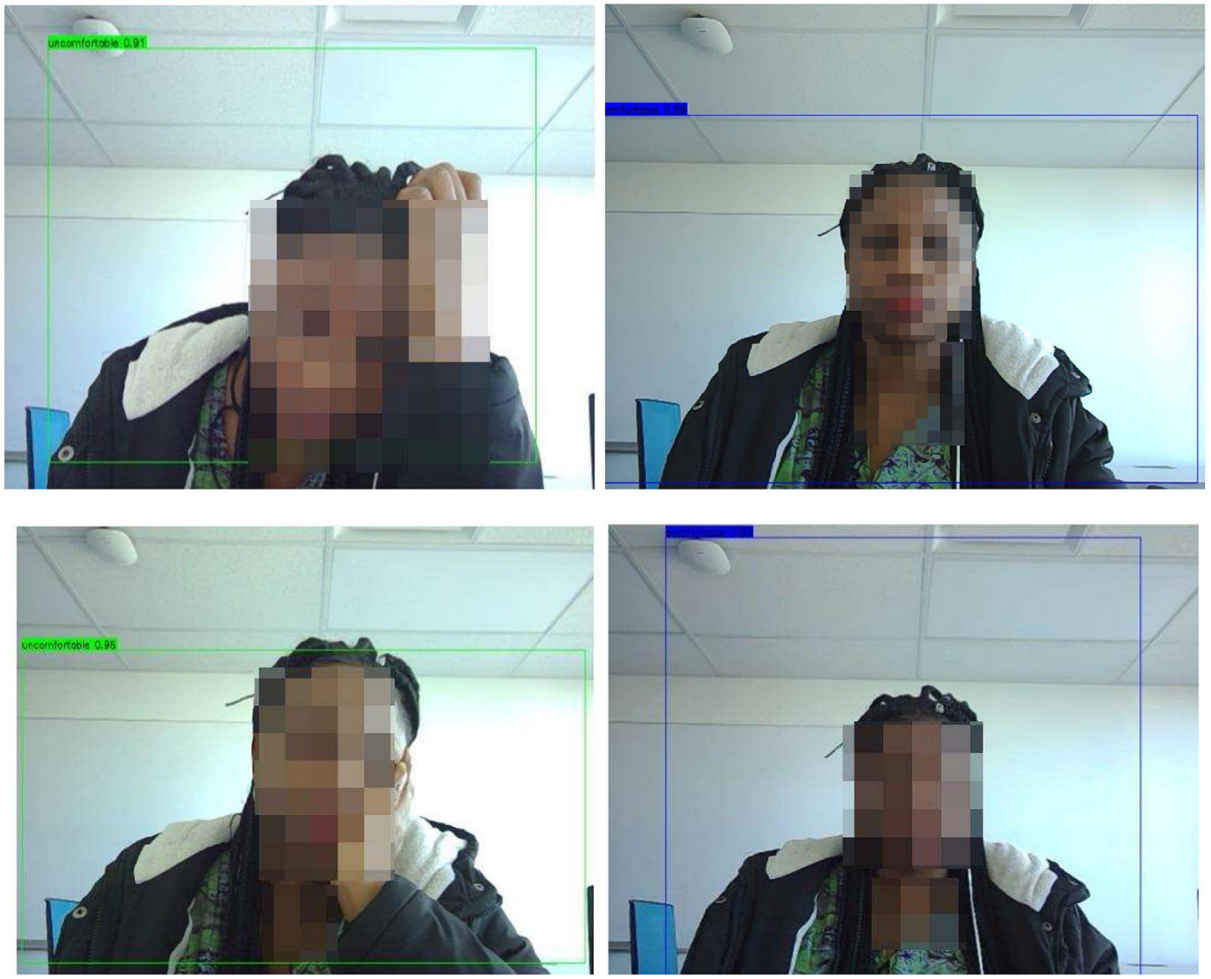
L-R: Yolo-V3-main model's posture detection: 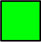 comfortable; 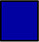 uncomfortable; 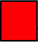 neutral.

We deployed the Yolo-V3-main model in real time, and the results indicate that the model performed significantly better in detecting both classes, “comfortable” and “uncomfortable” (refer to Figure 19). The Yolo-V3-main model achieved a mean average precision of 92% across 11 real-time test instances.

## 5 Discussion

The study explored design opportunities for persuasive systems based on real-time posture detection. We conducted two experiments, namely, the pilot and main studies, utilizing two deep learning algorithms: CNN and Yolo-V3. In this section, we discuss the results and propose design recommendations aligned with the overarching goal of the study, addressing how people can become conscious of their unhealthy posture practices in workplaces, whether sitting or standing. Furthermore, we relate these findings to answering the main research question: RQ: Can we design persuasive computers to detect unhealthy posture practices, such as sitting and standing, in workplaces?

From the pilot study, we observed that the CNN-pilot model tends to generalize its detection based on facial regions, occasionally extending to the neck regions. Additionally, for the CNN-pilot model, we reported on the detection of comfortable and uncomfortable postures with similar precision accuracy values. The lack of generalizability in the model raises concerns, particularly given our overarching goal of ensuring that persuasive technologies encourage people to maintain the right posture practices. It would be more suitable for individuals to be prompted to change their uncomfortable postures more frequently.

In contrast, the Yolo-V3-pilot model, with its anchor boxes, provided more comprehensive coverage and detection of postures. While it is common for Yolo models to generate multiple anchor boxes when detecting objects (Zhang et al., 2022), we observed trends of it detecting various body positions and regions associated with the required postures.

The main study results demonstrated a significant improvement in the CNN-main model compared with the CNN-pilot model. The convergence and drop of the loss values toward epoch 10 were notably pronounced, and the achieved mean average precision of 91% aligns well with the overarching goal of the study. The enhanced recognition of uncomfortable posture positions by the CNN-main model suggests that users of persuasive technologies would be more conscious.

Furthermore, there was a substantial improvement in the performance of the Yolo-V3-main model compared with the Yolo-V3-pilot model. The increased precision around both comfortable and uncomfortable body positions resulted in a mean average precision of 92%. Considering these results, we address the main research question by recommending the following.

D1. Persuasive systems can be customized to detect the posture positions of users. While there are promising prospects with the CNN model, particularly with additional training datasets, the Yolo-V3 model stands out in addressing crucial body positions such as the eyes, face, head, neck, and arms. The successes of Yolo-V3 models have been reported in real-time workplace monitoring, showcasing its capability to report multiple and significant positions (Saumya et al., 2020).

D2. Persuasive systems based on the Yolo-V3 model can be trained to recognize various environmental conditions, such as the lighting conditions of the room, desk height, and leg position of users. While previous study by Min et al. (2015) demonstrated the potential of using sensor reading based on back and arm movements, expanding to recognize more positions would necessitate multimodal datasets, sensors, and strategically positioned cameras to provide users with comprehensive feedback. It is important to note that this approach may require privacy permissions. The importance of aligning such feedback with users' privacy expectations, both in private and social spaces, has been emphasized in the study by Brombacher et al. (2023). Additionally, a study by Bootsman et al. (2019) was limited to reading lumbar (back) posture data, overlooking other key postures that directly impact the back, as we have reported (eyes, head, neck, and arms).

D3. Persuasive systems based on the Yolo-V3 model can be trained to provide auditory feedback to users, particularly benefiting individuals with visual impairments. This customization could involve real-world feedback systems, such as a single beep sound for correct posture positions and a buzzer sound for incorrect posture positions. To enhance usability, additional concepts may be implemented, such as helping users locate body positions through a screen reader. Feedback systems, as reported in the study by Brombacher et al. (2023), have been recognized as effective in capturing users' attention, especially when working behind a desk and receiving posture-related feedback.

### 5.1 The present study vs. related studies

We present our methodology and results compared with existing studies. Deep learning models, compared with SVM and other algorithms used in existing studies (Tang et al., 2015; Nath et al., 2018; Mudiyanselage et al., 2021; Zhang and Callaghan, 2021), capture the variability of highly complex patterns in datasets. Hence, while SVM performs significantly better with small datasets, deep learning models require a substantial number of datasets. In a related study (Mudiyanselage et al., 2021), SVM yielded 99.5% with 54 datasets for five weightlifting classes (10, 15, 20, 30, and 35 lbs.). The results from this study showed significant overfitting of the SVM model. In addition, in a related study conducted by Nath et al. (2018) with 9,069 datasets for three classes of ergonomic weightlifting risks (low, moderate, and high), SVM achieved ~80% accuracy.

We employed deep learning models (CNN and Yolo-v3) in this study, considering the variability of good and bad posture patterns that SVM and other non-deep learning models might not significantly capture. While deep learning requires large datasets, we report on our findings (Yolo-v3: 92% and CNN: 91% accuracy values using 2,265 posture images for two classes, good and bad) to propose future work with additional datasets. In another related real-time study by Zhang and Callaghan (2021) with different human postures (sitting, walking, standing, running, and lying) using deep learning multi-layer perceptron (MLP), the authors reported accuracy up to 82% with few datasets (30 training and 19 testing samples). Nevertheless, results from the study by Tang et al. (2015) revealed a significant number of misclassifications. Deep neural networks (DNN) in a similar task of human gesture recognition achieved an accuracy of 98.12%. This level of accuracy was attained using a dataset comprising 21,600 images across 10 distinct classes of hand gestures. While Yolo-v3 compared with CNN has not been explored in previous study, our results present the baseline performance of both models to guide future work.

### 5.2 Limitation of the study

While we report these significant findings of our study, we present the following limitations to improve future work. Though we found significant posture practices such as leg position and lying position, our findings are limited to the areas captured by the camera for sitting and standing body postures. Exploring these contexts further in future studies could inform the design of more wearable persuasive devices. In addition, our datasets are limited in size because there are a few instances of them publicly available. In the future, we will explore running experiments to collect additional ground truth datasets to enhance our model. In addition, to comprehensively assess the effectiveness of this technology in different workplaces (work-from-home, offices, and other spaces), a future study should include an evaluation of users' perceptions, considering both the advantages and disadvantages. We propose this framework as a valuable posture assessment tool which is applicable to any workplace setting, whether at home or in an office. Evaluating both contexts in future studies would contribute to a more comprehensive understanding of the applicability of technology. Finally, we had variations in the design of both models (YOLO-V3 and CNN); our comparisons might have favored YOLO-V3, especially with the dataset split ratio of 90% training and 10% validation sets. This is inconclusive at this point. We recommend that future studies explore setting the same standards for testing both models.

### 5.3 Implication of future design on system proximity detection and posture

**Figure 20:**
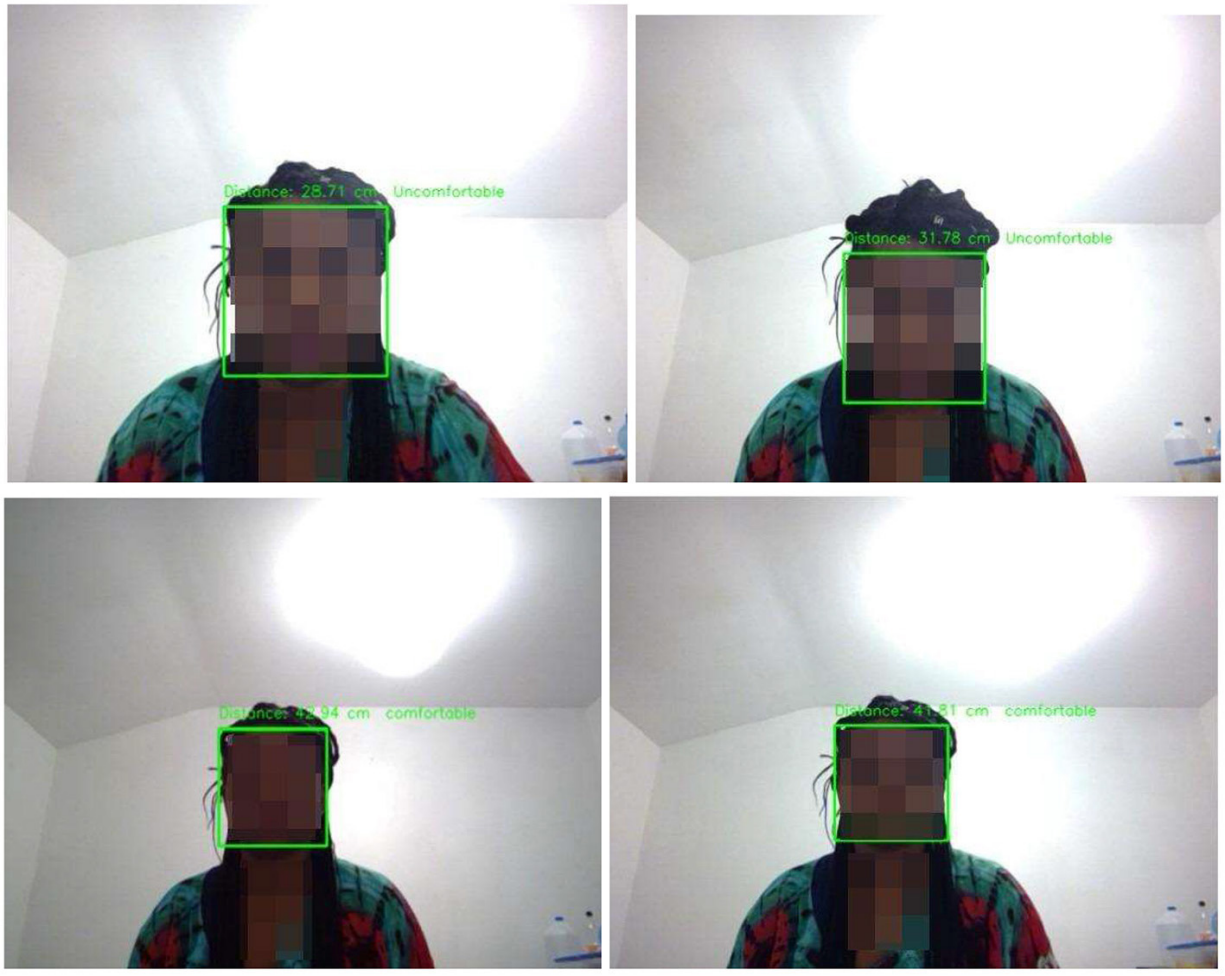
Proximity detection of uncomfortable and comfortable posture.

Considering the prospects of posture evaluation based on proximity detection, we designed a system to integrate with our proposed Yolo-V3 and CNN models in the future. It is recommended that a computer user maintain 40 cm from the computer (Woo et al., 2016). To meet this requirement, we modified the proximity detection program by Harsh Jaggi^11^ and presented the preliminary results, as shown in Figure 20.

## 6 Conclusion and future work

We explored potential designs for persuasive systems based on real-time posture detection. Given how significant persuasive systems and human factor engineering contribute to changing human behavior in workplaces, we conducted experiments using two deep learning models: convolutional neural networks (CNN) and Yolo-V3. These models have proven valuable in real-time detection of emotions, human activities, and behavior in previous research (Tan et al., 2021; Alsanad et al., 2022). Despite their effectiveness in various domains, little attention has been given to designing persuasive systems specifically for promoting proper postures in workplaces. Our overarching goal was to investigate how individuals can become more conscious of their posture practices while sitting and standing with a computer system. Additionally, we aimed to address the main research question: RQ: Can we design persuasive computers to detect unhealthy posture practices (such as sitting and standing) in workplaces?

Hence, based on the results of this study, we conclude with the following key insights:

1. Posture detection based on deep learning models would require a lot of datasets to implement.
2. Persuasive systems based on real-time posture detection should be tailored to capture more body positions. Overall, this helps to address more workplace requirements for behavioral changes.
3. There are prospects around eye strains, pupil datasets, and other contexts linked with stress. Hence, the framework of this study can be extended in the future.

In conclusion, our study highlights the potential for developing persuasive technologies that are specifically designed to support users in adhering to proper posture practices. The significance of this study prompts consideration for future exploration into themes such as more in-depth studies with large datasets, proximity detection, support for individuals with visual impairments in adopting optimal posture practices, eye strain detection, addressing various workplace requirements, and comparing outcomes of user studies with our technology from different workplaces such as work-from-home contexts, offices, and other ones.

## Data availability statement

The original contributions presented in the study are included in the article/Supplementary material, further inquiries can be directed to the corresponding author.

## Ethics statement

Ethical approval was not required for the study involving human data in accordance with the local legislation and institutional requirements. Written informed consent to participate in this study was not required in accordance with the national legislation and the institutional requirements.

## Author contributions

GA: Conceptualization, Data curation, Methodology, Writing – original draft, Writing – review & editing. RO: Supervision, Writing – review & editing.

## Footnotes

^1^ https://github.com/iwinardhyas/auto_annotation/tree/master/auto_annotatation

^2^ https://github.com/qqwweee/keras-yolo3

^3^ https://github.com/Abhishekjl/Facial-Emotion-detection-webcam-

^4^ https://keras.io/api/layers/pooling_layers/max_pooling2d/

^5^ https://keras.io/api/layers/core_layers/dense/

^6^ https://keras.io/api/layers/activations/

^7^ https://keras.io/api/layers/normalization_layers/batch_normalization/

^8^ https://keras.io/api/layers/activation_layers/leaky_relu/

^9^ https://keras.io/api/layers/reshaping_layers/up_sampling2d/

^10^ https://keras.io/api/layers/reshaping_layers/zero_padding2d/

^11^ https://www.linkedin.com/pulse/face-distance-measurement-python-haar-cascade-unlocking-harsh-jaggi
