# Supplementary figures and images for: Toward the design of persuasive systems for a healthy workplace: a real-time posture detection
Source: Front Big Data. 2024 Jun 17;7:1359906. doi: 10.3389/fdata.2024.1359906 (PMC11215059; doi:10.3389/fdata.2024.1359906)

**Supplementary Material**

**A1: YOLO-V3 Model Full Architecture**


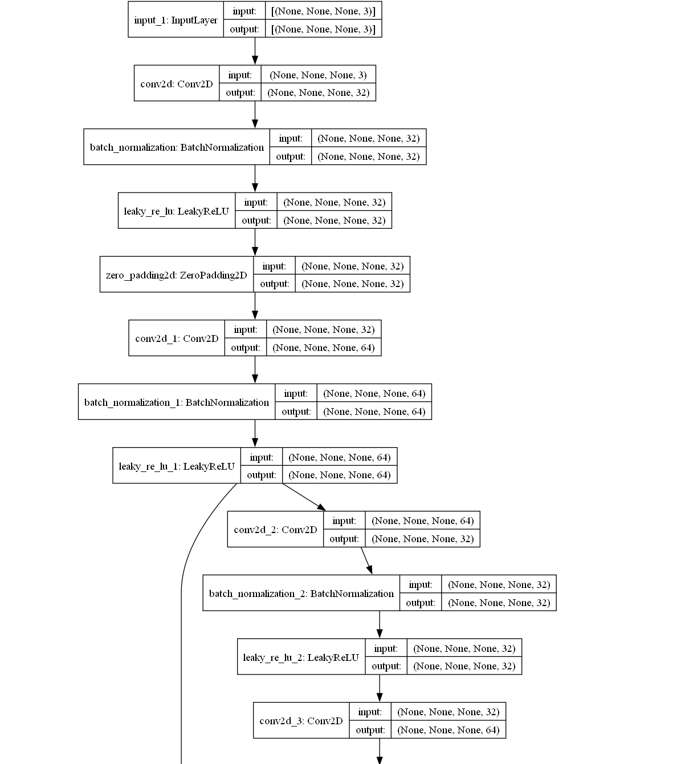


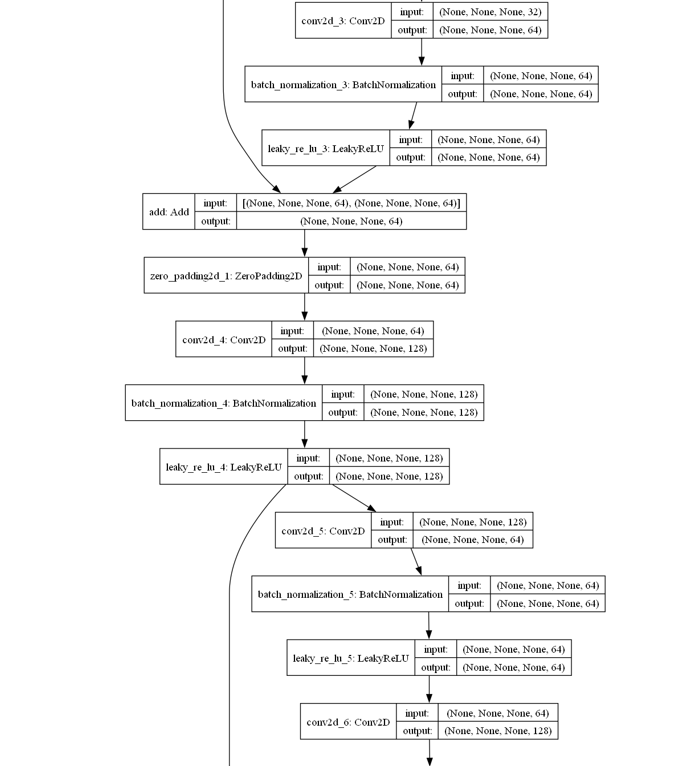


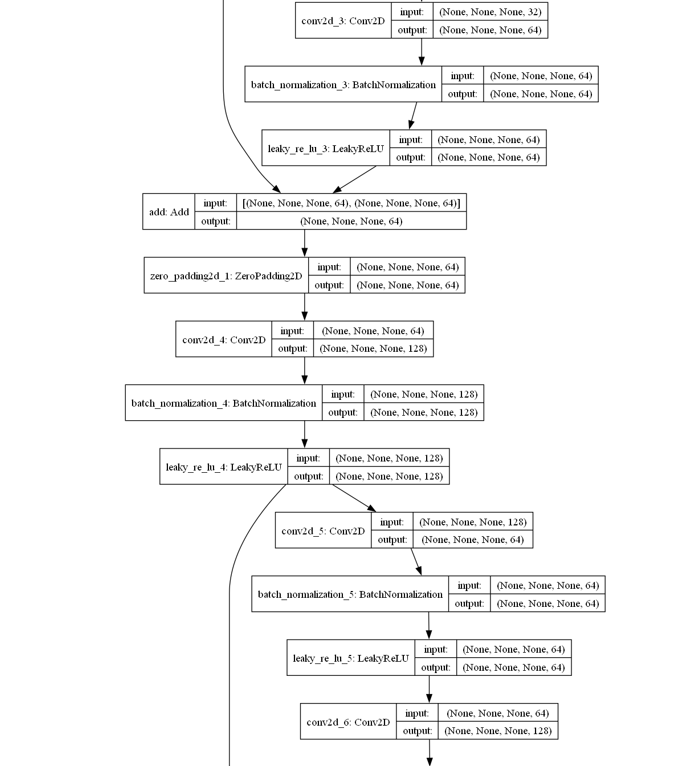


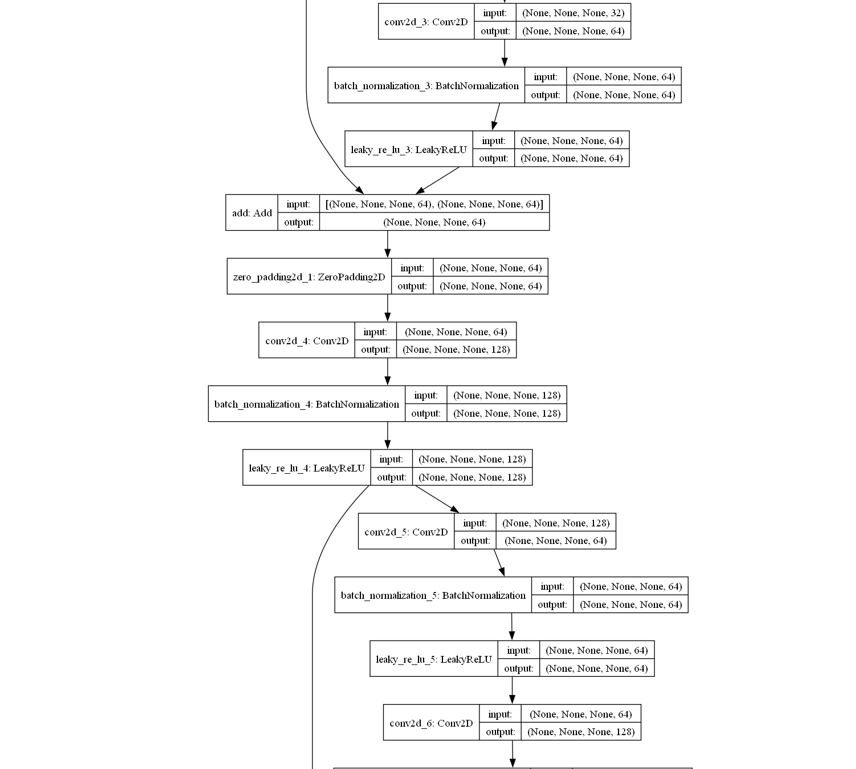


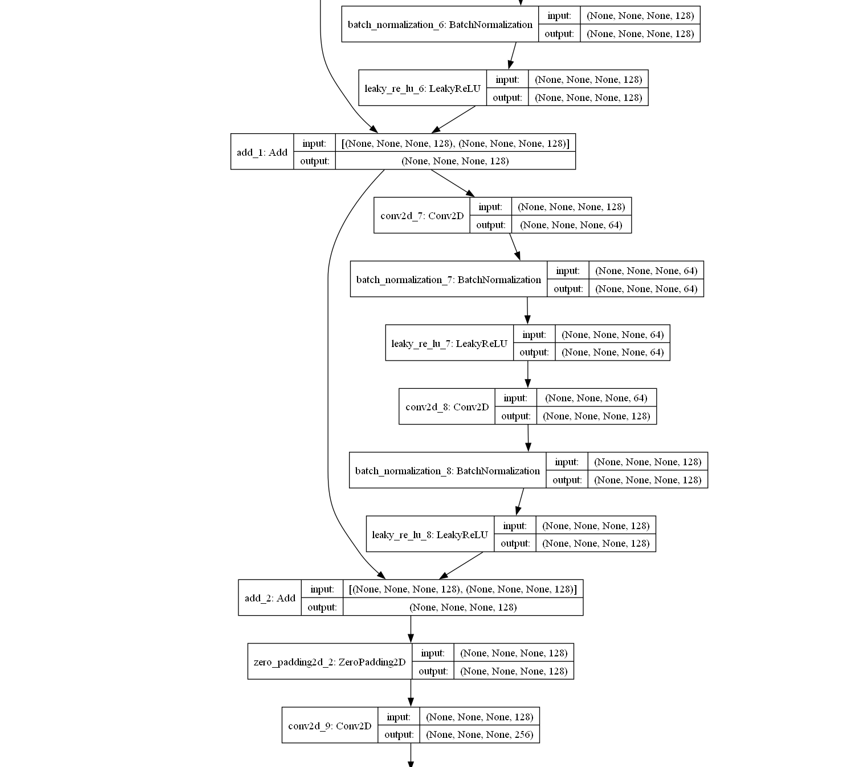


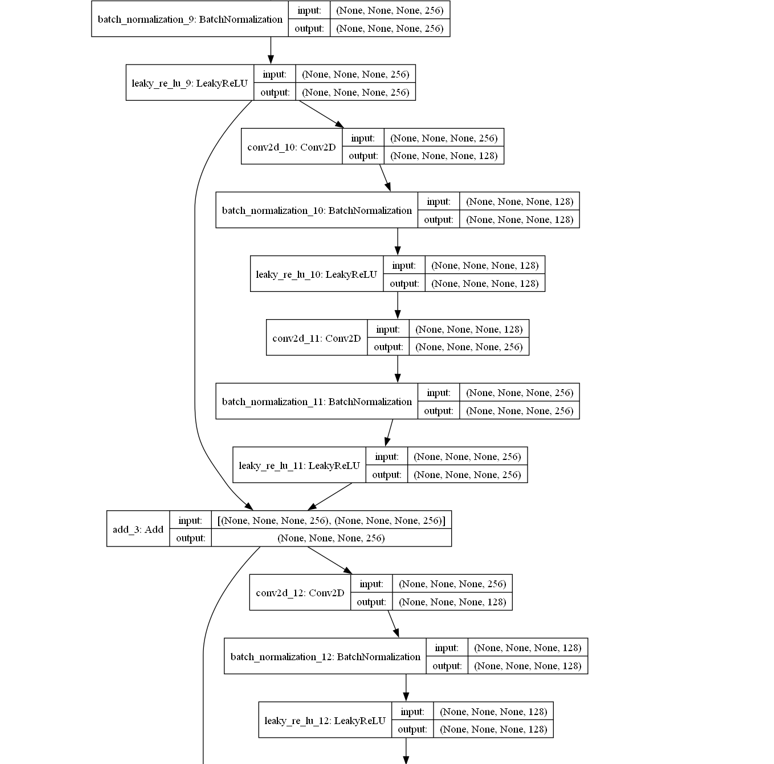


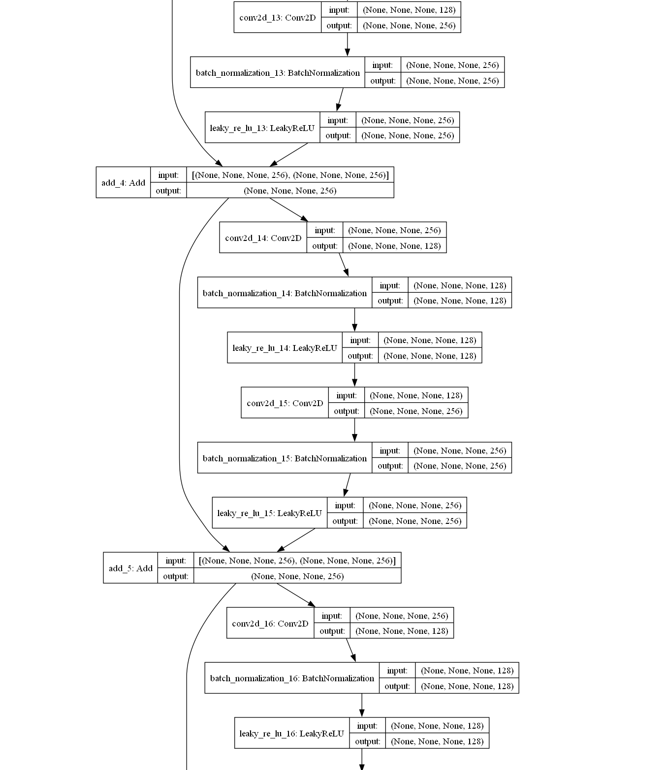


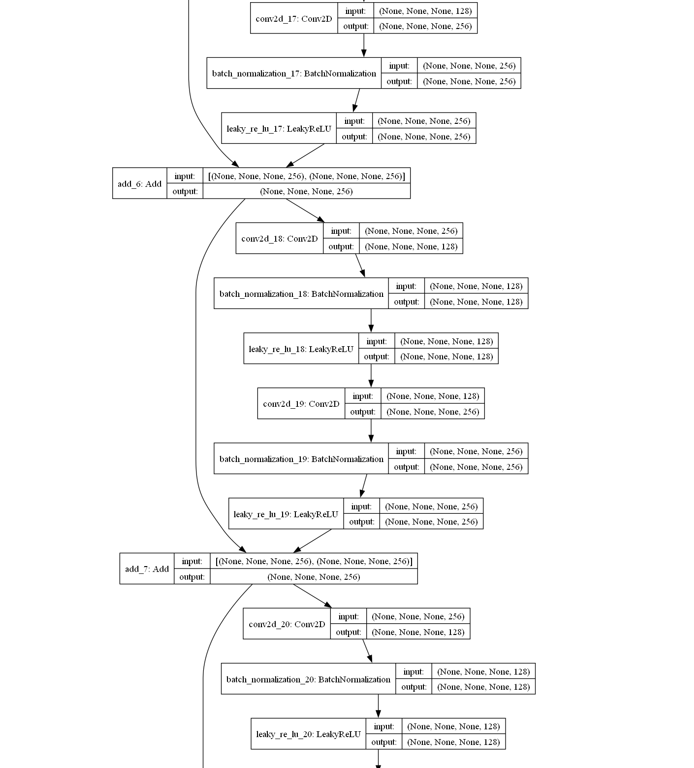


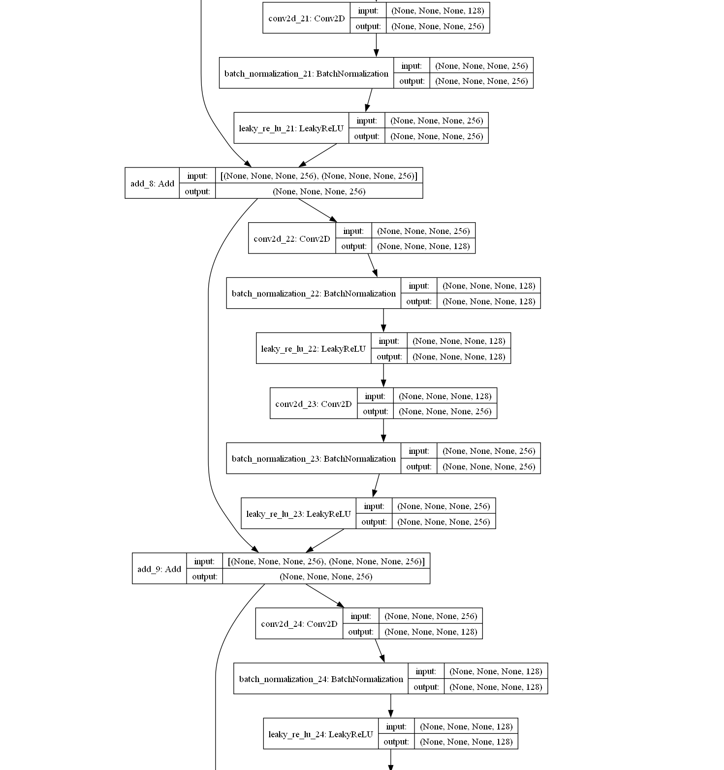


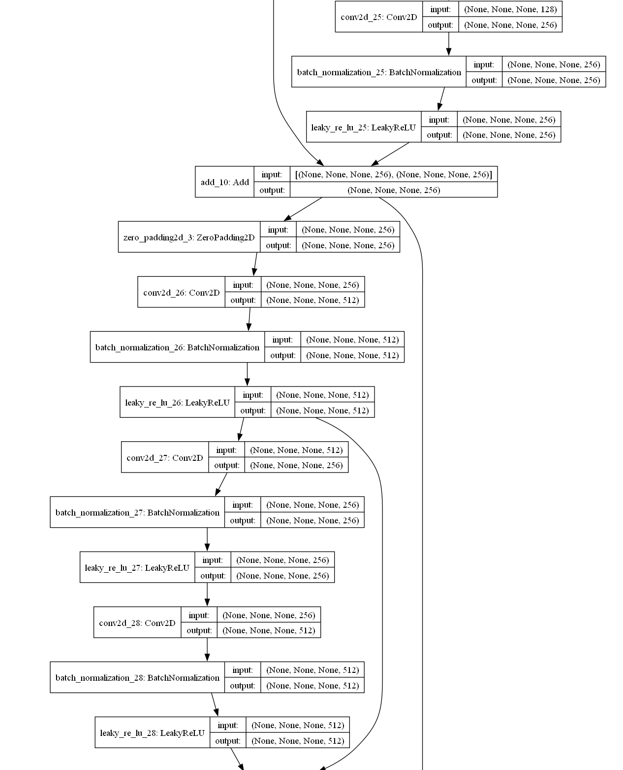


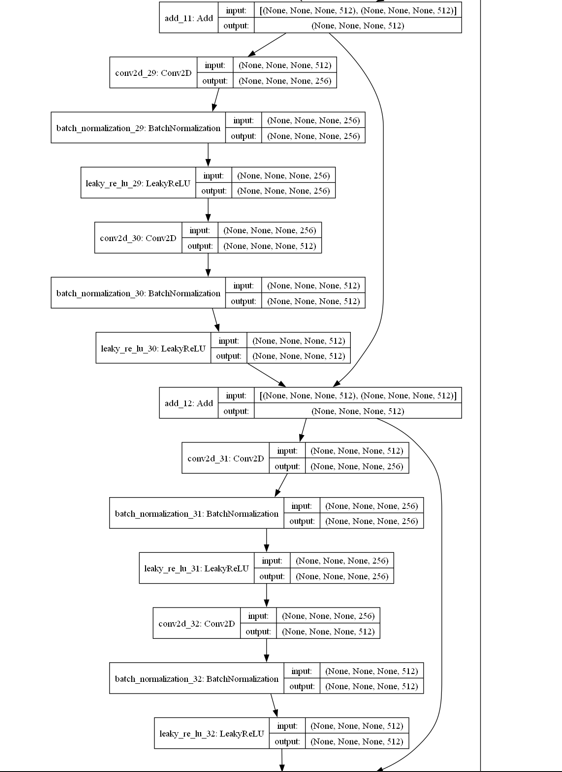


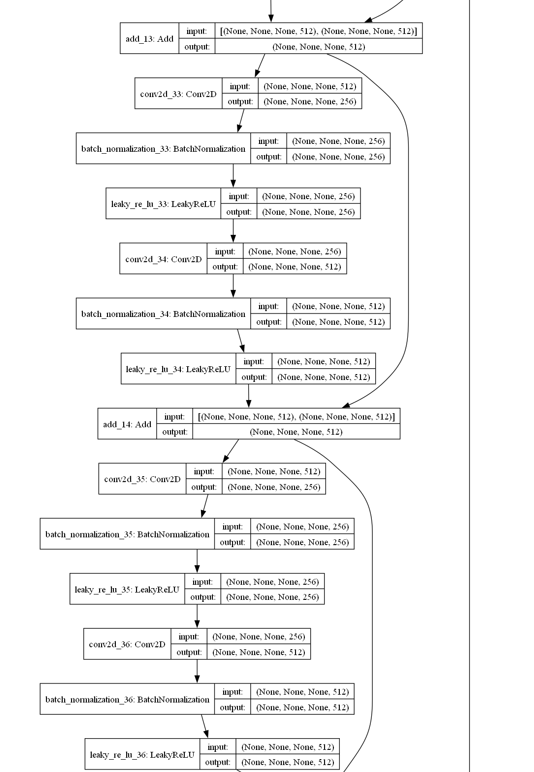


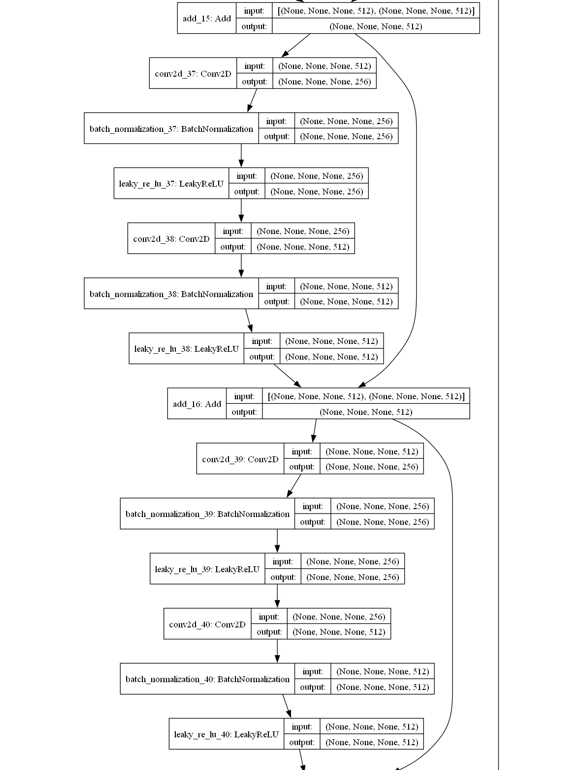


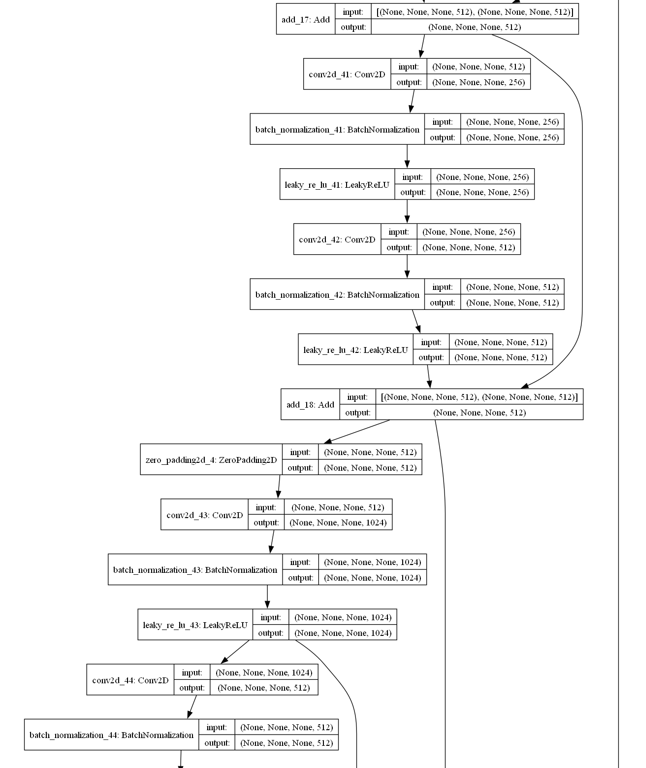


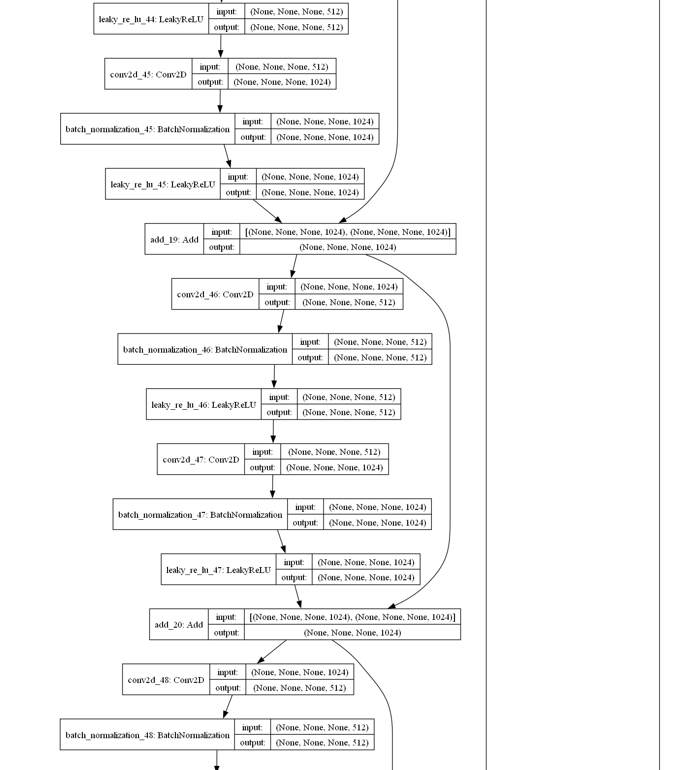


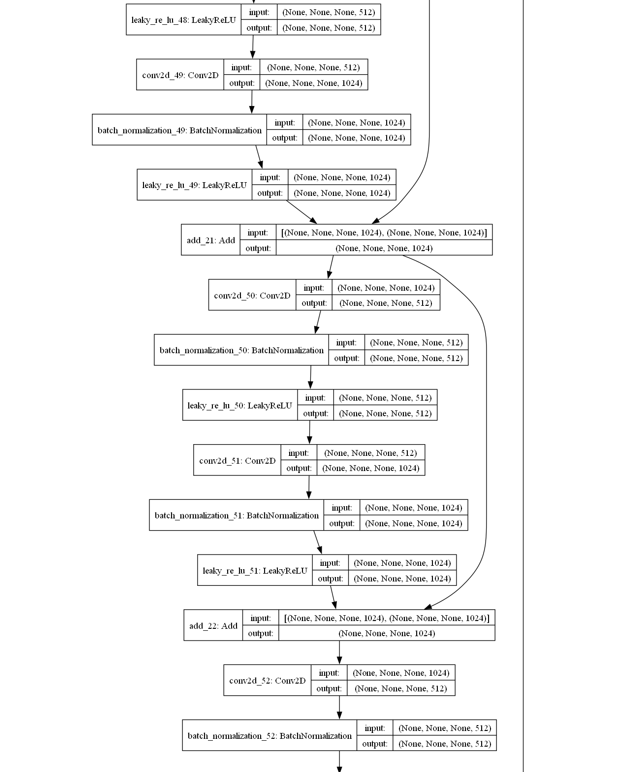


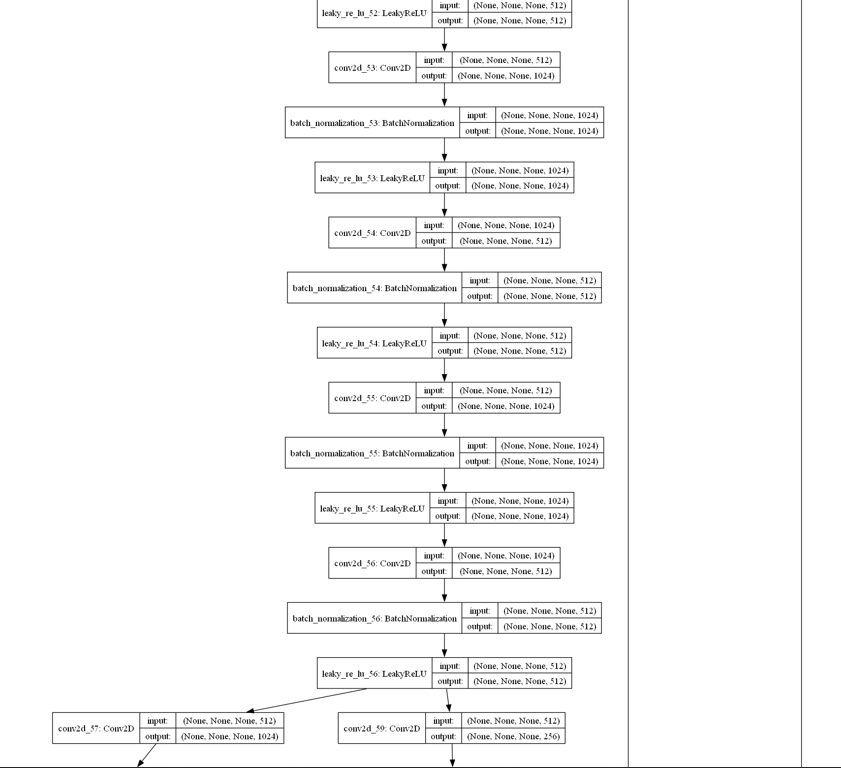


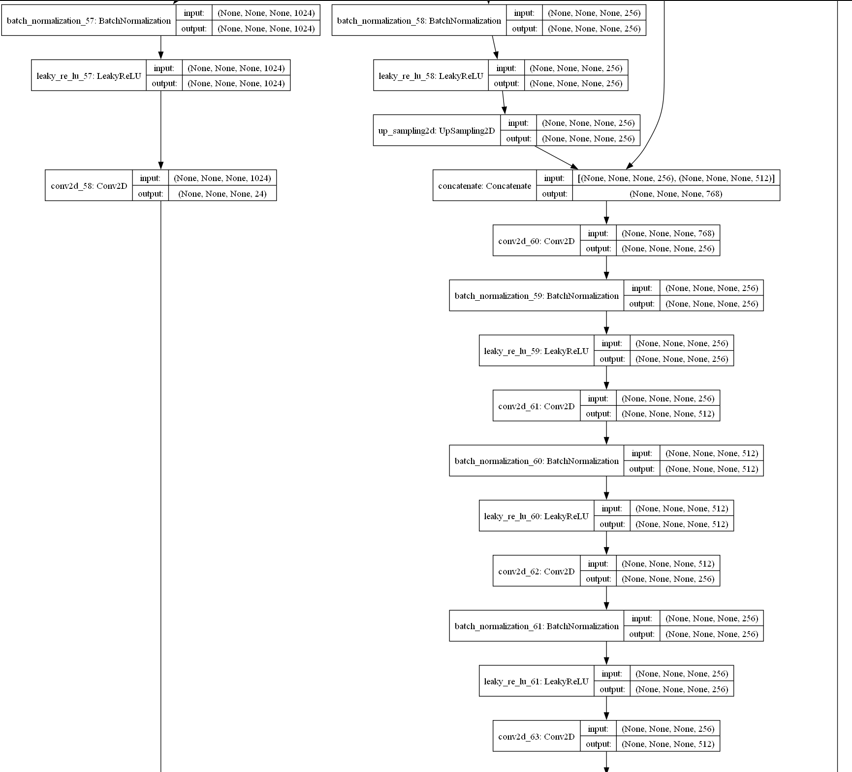


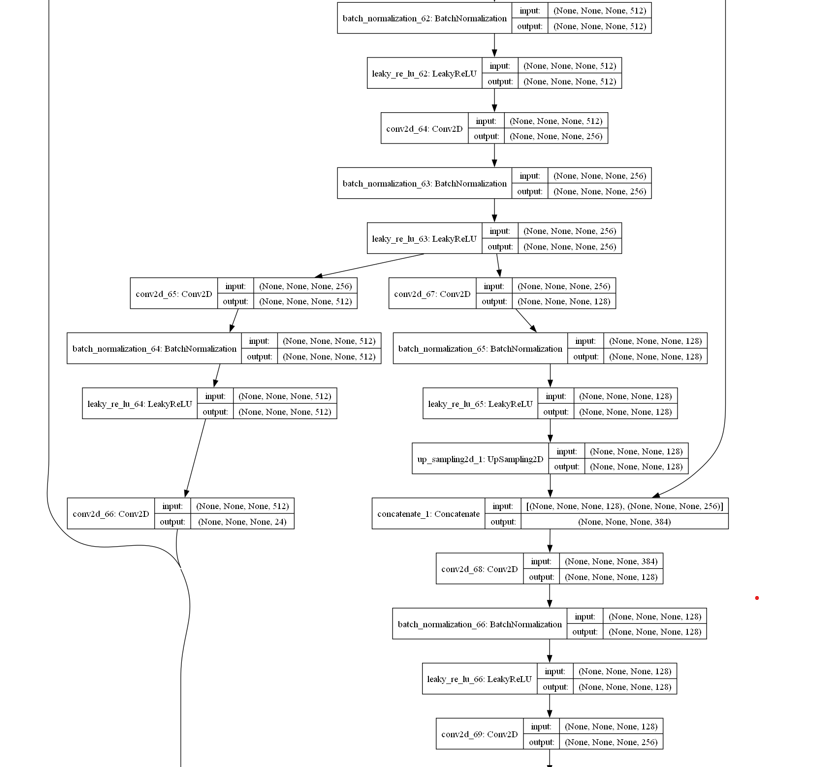


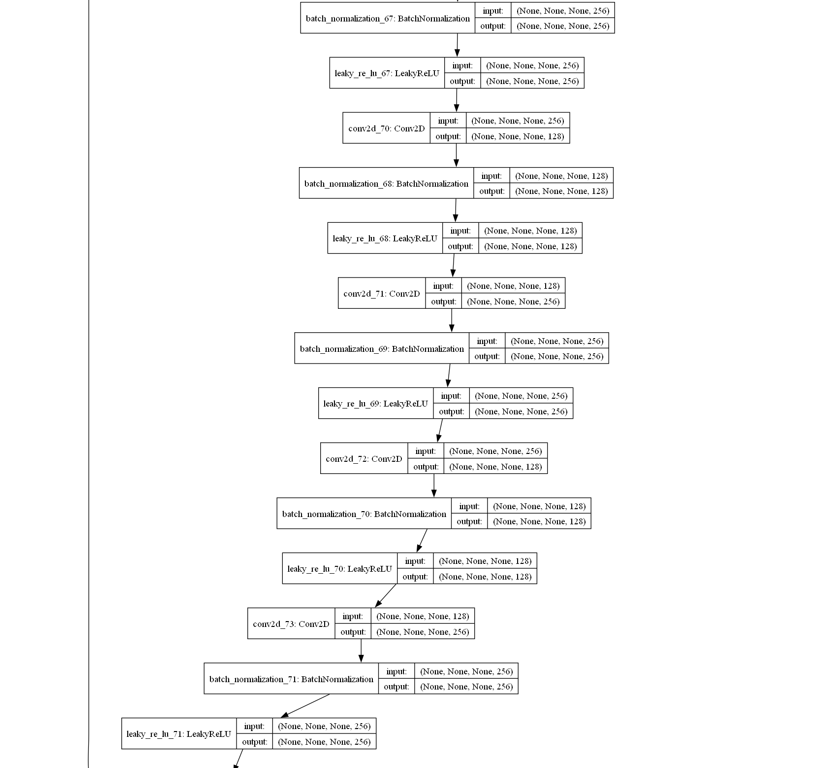


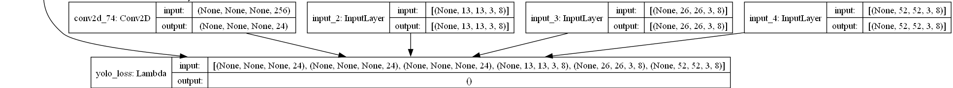

Supplement: Supplementary file 1 [file Data_Sheet_1.docx]
